# In-Depth Analysis of Low-Cost Micro Electromechanical System (MEMS) Accelerometers in the Context of Low Frequencies and Vibration Amplitudes

**DOI:** 10.3390/s24216877

**Published:** 2024-10-26

**Authors:** Piotr Emanuel Srokosz, Ewa Daniszewska, Jakub Banach, Michał Śmieja

**Affiliations:** 1 Faculty of Geoengineering, University of Warmia and Mazury, 10-957 Olsztyn, Poland; ewa.daniszewska@uwm.edu.pl; 2 Faculty of Technical Sciences, University of Warmia and Mazury, 10-957 Olsztyn, Poland; jakub.banach@uwm.edu.pl (J.B.); smieja@uwm.edu.pl (M.Ś.)

**Keywords:** low-cost MEMS accelerometer, low-frequency testing, detection of low amplitude vibrations, digital minimum threshold

## Abstract

Shock and vibration hazards to civil structures are common and come not only from earthquakes but most often from mining operations or foundation work involving the installation of piles using hammer-driving and vibrating technology. The purpose of this study is to present test methods for low-cost MEMS accelerometers in terms of their selection for low-amplitude acceleration vibration-prone object-monitoring systems. Tests of 24 commercially available digital accelerometers were carried out on a custom-built test bench, selecting four models for detailed tests conducted on a specially built precision vibration table capable of inflicting accelerations at frequencies of 1–2 Hz, using displacements as small as a few micrometers. The analysis of the results was based, among other things, on a modified method of determining the signal-to-noise ratio (SNR) and also on the idea of the effective number of bits (ENOB). The results of the analysis showed that among low-cost MEMS accelerometers, there are some that are successfully suitable for the monitoring and warning of excessive vibration hazards in situations where objects are extremely sensitive to such impacts (e.g., treatment rooms in hospitals). Examples of accelerometers capable of detecting harmonic vibrations with amplitudes as small as 10 mm/s^2^ or impulsive shocks with amplitudes of at least 70 mm/s^2^ are indicated.

## 1. Introduction

### 1.1. Background for the Genesis of the Research Topic

An earthquake is a phenomenon that occurs below the earth’s surface, but its effects are felt at the surface as one of the most deadly and devastating sources of hazards affecting local communities and the environment [1]. Most seismic phenomena are the result of natural stresses that develop at tectonic plate boundaries. The released energy then travels in the form of a shock wave (seismic wave), which causes sudden rupture and slippage along fault lines. Seismic activity can also be the result of human activity [2], so-called anthropogenic quakes. Seismic phenomena caused by mining tremors first began to be studied after a series of earthquakes in the vicinity of a gold mine near Johannesburg (South Africa) in 1894 [3]. Since then, additional types of human-induced seismicity have been unraveled, including earthquakes associated with groundwater extraction [4], oil and gas extraction [5,6] deposit dumping and fluid injection [7], and hydroelectric dam construction [8]. Determining whether a given earthquake is natural or induced is not easy, since those recorded on the seismogram look very similar. Seismic records generated by seismographs determine the location, depth, and, most importantly, magnitude of a seismic event. As Ramirez-Zelaya et al. [9] point out, many sensors of this type have certain limitations, most often concerning the measurement of displacements (land surface) in higher frequency bands. Lei Li et al. [10] state that with traditional methods, measuring accurate microseismic velocity inversion is challenging due to the low signal (useful)-to-noise ratio of field data.

“Sources of vibrations” can also be dynamic loads from railroad [11] and road traffic [12], the operation of construction equipment especially with vibration effects (slabs and rollers), machinery (hammers, presses), explosions [13], blasts [14], and collision events. Ground vibrations caused by rock mass shaking, but also vibrations generated during pile driving [15], propagate through the ground and can result in damage to surface structures (buildings, bridges, tunnels) and vibration-sensitive equipment. The noticeable increase in the requirements for providing protection against vibrations of buildings and people in them, as well as the observed steady increase in both the number of vibration sources and their intensity, have contributed to the inclusion of such impacts in the design procedure and diagnosis of buildings [16,17]. Criteria for the evaluation and control of dynamic influences have been included in standards (e.g., [18,19]). The standard in [20] recommends that “inspections should be carried out at the design, execution and maintenance stages”. Sensors are installed at selected locations in the structure to record changes in physical values relevant to the safety of the facility, tunnel, or rock mass. The installation of structural measuring devices on a facility should be due not only to the need to meet the relevant construction law but primarily for reasons of safety in the implementation of construction work (not allowing failure or disaster) and the continued operation of the existing facility without exposing users to unreasonable costs [21].

### 1.2. MEMS Accelerometer Applications in the Context of Safety Monitoring

In 2010, a strong earthquake occurred in Darfield, New Zealand, after which the use of nearly 200 low-cost micro-electromechanical accelerometers was immediately deployed [22]. Electromechanical microsystems [23] are a collection of mechanical, electronic, and electrical devices that are highly miniaturized and integrated with each other. In a way, they are “smart” systems that combine electronic and mechanical components operating in a small space. The use of MEMS (Micro Electromechanical Systems) in seismology and related fields is relatively new. In addition to applications strictly related to earthquake observation and monitoring, MEMS sensors are also used for seismic research and imaging, vibration monitoring, and diagnostics of building structures [24,25,26]. Traditional seismic sensors are analog, based on the principle of spring-mass to achieve high-precision measurements, but they also have a large mass and are heavy, making them bulky and difficult to transport and use. They are also usually very expensive. On the other hand, there are miniaturized sensors that are easy to carry, install, and maneuver. The advances made in MEMS technology have opened the door to new applications in the broad field of seismology with innovative solutions. MEMS accelerometers, of course, have some shortcomings. The team of Scudero et al. [27], in addition to the numerous advantages, list the disadvantages of microsensors, including the noise level, which in the best accelerometer is about −100 dB. Another disadvantage is the relatively poor response at low frequencies, suggesting that the measurement of strong seismic movements is more suitable for MEMS sensors. In a technical note [28], we find the following notation: “Traditional broadband seismometers and tiltmeters do not have the range to measure strong seismic events and traditional strong motion sensors do not have the sensitivity or stability to make good long-term geodetic measurements. Quartz Crystal Triaxial Accelerometers, with parts-per-billion resolution, can measure strong earthquakes without clipping and can use the invariance of Earth’s 1 g gravity vector as a reference for long-term measurements”. However, MEMS technology is evolving so rapidly that improved versions of products are constantly being released to better meet the technical requirements for optimal earthquake monitoring.

A comprehensive review of the various applications of MEMS accelerometers, including with particular emphasis on their suitability for vibration monitoring, can be found in the review paper by Binali et al. [29]. Attention is drawn to the fact that the analysis of vibrations recorded by MEMS sensors is very useful in monitoring the condition of buildings or machine components, as it successfully enables the detection of the causes of the most common failures or defects. MEMS accelerometers are becoming an essential component of systems for predicting and monitoring the condition of engineering structures, thus preventing unexpected failures and costly repairs. Low-cost sensors of this type have already found practical applications for monitoring the technical condition of buildings—Lin et al. [30] demonstrated the usefulness of MEMS accelerometers using the example of observation of a public building in constant operation and located in an area where periodic earthquakes occur. The observation system was characterized by low-cost MEMS accelerometers with low intrinsic noise that were capable of recording low-frequency, low-amplitude dynamic responses.

The commercialization of MEMS accelerometers typically focuses on minimizing their manufacturing costs, followed by optimizing their power requirements, and finally their reliability. In the case of MEMS accelerometers with a design solution dedicated to seismic issues, the problem of achieving a sufficiently low noise level (i.e., 1 μg/√Hz) remains a major technological challenge [31]. Current commercially available accelerometers of this class are expensive but can function as short-period seismometers for measuring ground vibrations with frequencies in the range of 0.1–10 Hz and even as broadband seismometers recording seismic signals with frequencies starting as low as 0.01 Hz [32]. Many publications address the problem of determining the static characteristics of accelerometers. However, the dynamic characteristics are more interesting from the point of view of monitoring vibrations that threaten engineering structures. Wang et al. [33] presented the problem of determining the performance of accelerometers under complex dynamic loading circumstances. They compared a standard capacitive accelerometer with an accelerometer constructed by their own design, proving that it is suitable for measuring low-frequency vibrations in the 0.5–5 Hz range. It should be noted that before MEMS technology was applied to seismology, there were colossal differences between geophone and seismometer/seismograph type devices, both in terms of the idea of operation, design and the resulting detailed applications. While seismometers are typically designed to detect extremely small ground displacements, with frequencies ranging from 0.01 to 50 Hz, the frequency bandwidth of geophones is usually in the range of 1–15 Hz [26]. The development of design solutions for digital geophones, whose shock-sensitive element is a MEMS sensor, has made it possible for them to record vibrations containing an extremely wide frequency bandwidth reaching as high as 500 Hz [34], which has meant that the limit of possibilities and applications separating geophones and seismometers is no longer clear.

There is no doubt that the observation and registration of vibrations having natural sources as well as those originating from human activities is an important and current topic of scientific research. However, the literature lacks test procedures and interpretive methods for evaluating the suitability of MEMS accelerometers for recording low-frequency oscillations with maximum amplitudes that lie at the limit of their intrinsic noise. This paper presents the results of a study of 24 accelerometers, analyzing their properties with a view to the feasibility of designing and implementing portable and low-cost devices for monitoring dynamic impacts on building structures. Special emphasis was placed on testing procedures and the interpretation of results in the context of evaluating the utility of the selected MEMS accelerometers in low-frequency and low-amplitude vibration monitoring systems for the safe operation of particularly sensitive building structures, such as hospitals with surgical operating rooms.

## 2. Materials and Methods

The in-depth analysis, which is based on specially developed equipment and test procedures, consists of two main stages:

- Initial selection of sensors based on an assessment of the stability of their readings;
- Analysis of selected sensors in terms of the minimum acceleration amplitude threshold that can be correctly identified and interpreted by algorithms running in embedded systems.

### 2.1. Stage I: Stability Testing

#### 2.1.1. Selection of MEMS Accelerometers for Stability Testing

Twenty-four commercially available digital MEMS accelerometers were selected for this study—a summary of their most important parameters is presented in Table 1. It should be noted that some of them, despite their long existence in commerce and constantly emerging new technological solutions, are constantly in high demand among engineers/designers of various types of IMU systems (e.g., ADXL345, MPU6050, etc.). It is obvious that the presented selection of accelerometers may seem biased, but the authors assure that they were guided by total “randomness” when purchasing these devices, and the only criterion was “off-the-shelf” availability confirming the popularity of the technological solutions they represent.

#### 2.1.2. Method and Experimental Setup for Stability Testing

One of the elements of great importance in terms of quality assessment of the MEMS devices under study is the noise characteristics of the sensory systems (random stray, constant error stability), which is most often determined in the form of Allan variance (AV). Originally, this algorithm was designed to describe the accuracy characteristics of clocks, but it has been successfully used to determine the errors of any time-correlated measurements [35,36,37,38]. The method involves presenting the average drift errors of a system as a function of time averaging. Currently, the Allan variance is an IEEE 952-1997 (Institute of Electrical and Electronics Engineers Standards Association) standard used to determine the accuracy characteristics of gyroscopes [39]. The algorithm consists of the following steps: collecting data from a long period of time; dividing the collected data into equal-length samples; averaging the data in individual parts, thus creating a so-called list of averages; and calculating the Allan variance value from the following formula [38]:

(1) $$AV\left(\tau \right)=\sigma^{2}\left(\tau \right)=\frac{1}{2\left(n-1\right)}\sum {\left({a\left(\tau \right)}_{i-1}-{a\left(\tau \right)}_{i}\right)}^{2}$$

where *τ*—averaging time, *a*(*τ*)—average value for one sample, and *n*—number of parts averaged.

**Figure 1:**
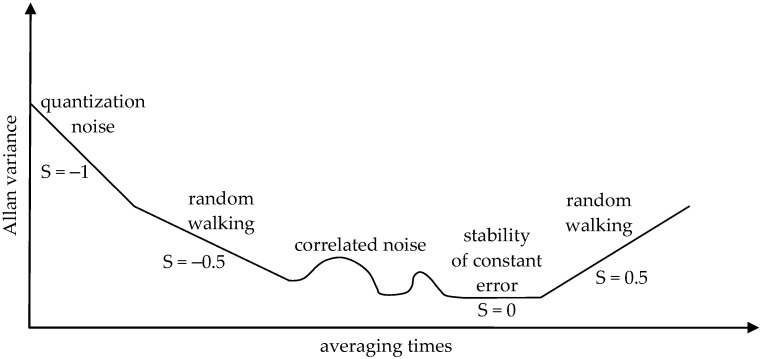
Allan variance interpretation (S—slope factor) [38].

The selection of time *t* to be used in the analysis depends on the device under study. In the case of inertial sensors, the selected averaging times range from a few tens of seconds to several hours. In order to correctly read the quantities of interest, the results of the calculations are presented on a graph on a vertical logarithmic scale. On the vertical axis are the values of the Allan variance, and on the horizontal axis are the averaging times. An example of the shape of the AV graph and its interpretation is shown in Figure 1 [38].

In the case of MEMS accelerometers, the value sought for the random stray velocity can be read from the AV plot at an averaging time *t* equal to one hour, when the plot has a slope *S* = −1/2. The stability value of the mean error, on the other hand, can be read for an averaging time of about 100 s. (depending on the quality of the sensor) at the lowest point of the plot, where the slope is *S* = 0 [39]. When measuring to determine random straying, the length of the measurement session is very important. It is assumed that for the correct determination of random stray, the minimum length of the session should be 9 h [36]. The stability of the constant error is read for shorter averaging times because the error occurs cyclically over short periods. For larger averaging times, the impact of stability is so small that it is lost in the random straying of the device. Despite this, an extended measurement session time of a minimum of 24 h was adopted in the completed test plan.

**Figure 2:**
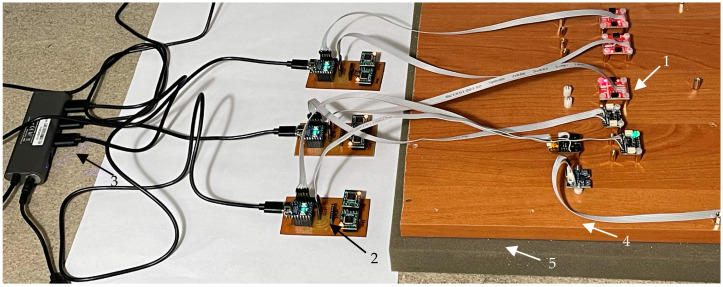
Experimental setup for AV measurements. 1—accelerometer; 2—dual-channel data acquisition unit; 3—power supply; 4—wooden board; 5—polyurethane foam. The resonant frequency of the system as a mass-spring model is about 44.7 Hz.

In the first stage, samples of readings from all sensors were collected, assuming the most common minimum operating range of ±2 g and the maximum available resolution of AD converters. Measurements were made at a frequency of about 100 Hz for a period of time of at minimum 24 h. The experimental setup consisted of a wooden board with dimensions 800 mm × 300 mm × 18 mm, to which sensors were attached with screws. The board rested on 30 mm thick polyurethane acoustic foam insulating it from the floor (Figure 2). The measurements were carried out in the UWM laboratory located in the basement of a building on the outskirts of the university campus in order to eliminate environmental influences as much as possible (constant temperature and humidity, no vibrations caused by traffic, human activity, etc.). During the tests, attention was paid to details such as proper shielding, low-noise power supply, galvanic isolation of systems powered by boost converters, and linear regulators.

**Figure 3:**
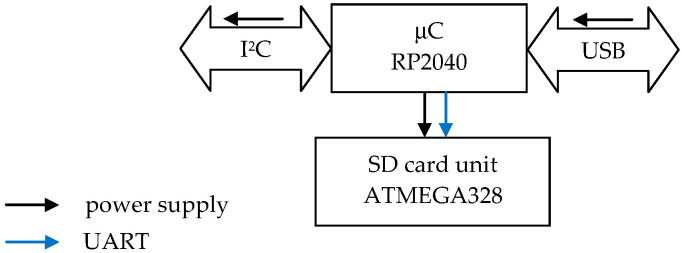
Block diagram of a single MEMS sensor data acquisition unit.

The sensors were controlled via the I^2^C bus (data rate 400 kHz) using a dual-core RP2040 32-bit microcontroller (one core handled transmission with one accelerometer, Figure 3). The data, in the form of a stream with a fixed sampling rate of 100–125 Hz (depending on the accelerometer model, see Table 2), were sent via a UART port to a prefabricated module managed by an 8-bit ATmega328 microcontroller (Microchip), which stored it on an SD card via an SPI port.

### 2.2. Stage II: Determination of Identifiable Minimum Amplitude of Low-Frequency Vibrations

#### 2.2.1. Selection of MEMS Accelerometers for Stage II Testing

The second parameter characterizing MEMS sensors, after stability, assumed to be of utmost importance in this discussion, is the minimum threshold resulting from the instantaneous noise values and the resolution of the sensor itself and possibly the on-chip analog-to-digital converter system. Taking into account the fact that the sensors in question are tested in terms of their suitability for the construction of low-cost devices for monitoring building structures exposed to dynamic impacts, the possibility of extracting a useful signal from noise is the basic evaluation criterion.

Selecting the best sensor based on AV analysis is not a simple task, as it depends primarily on the significance of the impact of individual errors expressed by the variance on the measurement performed in specific applications. Given that the Allan variance can help determine the ideal observation time for a particular measurement, and this study focuses on short-term measurements, the representative part of the AV is its values located in its initial fragments that visualize quantization noise. The tested sensors were classified into three groups/grades, arbitrarily adopting the following values for the maximum AV value corresponding to quantization noise (for τ < 2, denoted here as AV_2_): AV_2_ < 10^−6^ (grade A), 10^−6^ ≤ AV_2_ < 10^−5^ (grade B), and AV_2_ ≥ 10^−5^ (grade C). It should be noted that, despite the sharp criterion adopted, MEMS sensors with very different parameters were selected for stage II analyses to objectively visualize the performance of the proposed test method and interpretation of obtained results.

#### 2.2.2. Method and Experimental Setup for Stage II Testing

**Figure 4:**
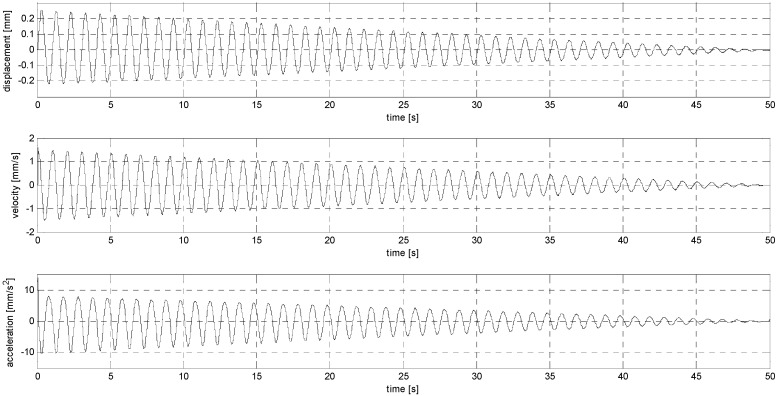
Harmonic signal with linearly decreasing amplitude (S1).

**Figure 5:**
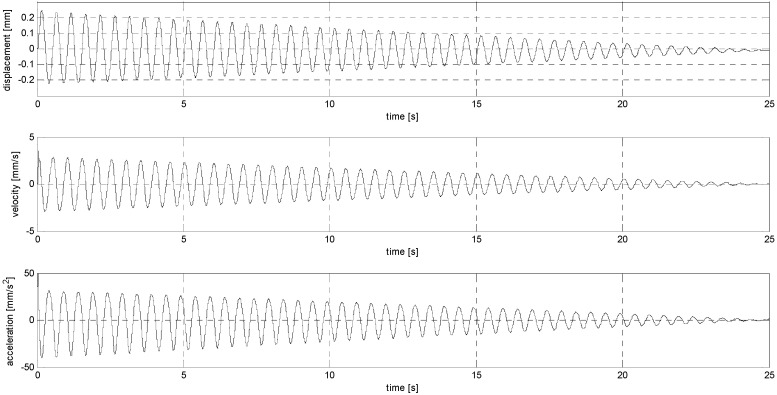
Harmonic signal with linearly decreasing amplitude (S2).

**Figure 6:**
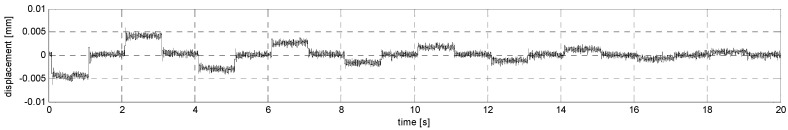
Step signal with linearly decreasing amplitude (P1).

**Figure 7:**
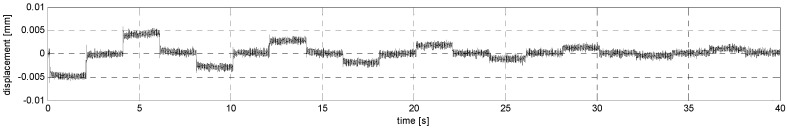
Step signal with linearly decreasing amplitude (P2).

The main idea of the experiments conducted in the second stage of the research was to subject the sensors to harmonic and impulsive vibrations. In the first case (sub-stage S), the sensors were loaded with sinusoidal vibrations at frequencies of 1 and 2 Hz (sub-stage S1 and S2, see Figure 4 and Figure 5), with the amplitudes of the loads not being constant but fading linearly to zero, generating accelerations with values starting at 10 mm/s^2^ and 40 mm/s^2^, respectively. In the second case (sub-stage P), the sensors were subjected to pulse loads with a maximum displacement amplitude of 0.005 mm and durations of 1 and 2 s (sub-stage P1 and P2, see Figure 6 and Figure 7). Displacements were induced alternately with the amplitude being reduced by 0.001 mm in each successive loading cycle (i.e., 0, −0.005, 0, +0.005, 0, −0.004, 0, 0.004, 0, etc.). A summary of the sensor load parameters is included in Table 3.

The goal of sub-stage S is to identify the minimum amplitude of low-frequency vibrations that can be recognized in the waveform recorded by the sensors. The maximum amplitude of 40 mm/s^2^ was chosen so that it is below the threshold required for objects that are particularly sensitive to vibrations (e.g., surgical operating rooms in hospitals—according to Polish regulations, the maximum amplitude of acceleration must not exceed 50 mm/s^2^ in their structures). In turn, the aim of sub-stage P of this study is to check the response of the sensors to impulsive loads having the characteristic of a wide spectrum of frequencies in terms of the possibility of recognizing a momentary disturbance against the background of equipment noise. It should be noted that in the case of dynamic impacts on civil structures, the two above always occur in the form of an individual combination with different mutual proportions of participation in a specific impact occurrence. Additionally, readings from the sensors were carried out at a constant frequency of 400 Hz, which made it possible to check the suitability of the sensors for detecting sudden changes in their position at relatively low sampling frequencies (the influence of the inertia of the entire system but also of the internal structure of the MEMS). It should be emphasized that sampling frequencies in the range of 200–500 Hz are most often used in systems for long-term monitoring of dynamic impacts on buildings (e.g., mining damage). Sensors that successfully pass both types of tests, i.e., can unambiguously identify impacts at both extremes, will be able to be indicated as potentially applicable to practical low-cost monitoring systems for building structures.

The following procedure is proposed for interpreting the results of sub-stage S:

- Extracting the signal from the raw data using a low-pass filter with an assumed cutoff frequency (cleaned signal);
- Determination of the noise based on the difference of the signals: raw and cleaned;
- Determination of the raw signal-to-noise ratio (SNR);
- Determination of the ratio of the cleaned signal to the noise (modified SNR, MSNR);
- Determination of the effective number of bits based on the SNR (ENOB);
- Determination of the threshold amplitudes based on the ENOB and MSNR.

Interpreting the results of sub-stage P in a manner analogous to sub-stage S is much more difficult and introduces a lot of ambiguity due to the nature of the forcing signal. Therefore, the evaluation was made by adopting only the simplest criterion of the occurrence of a reading with a value exceeding the assumed threshold, which is commonly used in systems that support tap and double-tap detection.

**Figure 8:**
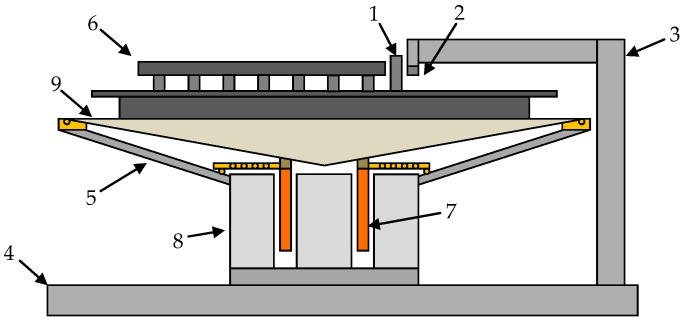
Exciter scheme (modified 200 W wideband loudspeaker). 1—NdFeB magnet; 2—hall sensor; 3—aluminum support frame; 4—base (steel ballast braced with plaster); 5—steel frame; 6—accelerometer mounting platform (plastic composite covered with shielding copper); 7—copper coil; 8—ferrite magnet; 9—loudspeaker diaphragm.

The sensors selected in the previous stage were mounted on a laminate table and shielded by a copper layer of a specially designed and constructed device—a low-frequency, low-amplitude shaker/exciter—to determine the minimum acceleration value that can be successfully recorded by these sensors (Figure 8). This dedicated test stand consists of four basic components: an electromagnetic exciter for mechanical vibrations, a control signal generator, a measurement data acquisition system, and software installed on a laptop computer.

**Figure 9:**
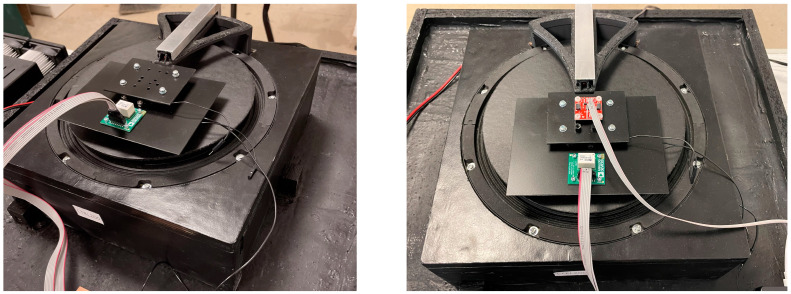
Vibration table with various MEMS placement configurations.

The electromagnetic exciter was equipped with a set of mounts matching the PCBs with the accelerometers under test (Figure 9). The most important parameters of the exciter are summarized in Table 4.

In order to control the generated displacement waveforms as accurately as possible, the bench consists of individually selected components, both mechanical and electronic. The design of the electrodynamic transducer, which is the exciter of the vibrating table, is based on a broadband speaker capable of carrying a constant current of 2 A without the risk of thermal damage (typical bias current did not exceed 0.5 A). It should be noted that the design of the test bench was modified and improved several times in order to best adapt its parameters to the adopted research plan and the properties of the MEMS accelerometers used for analysis. Attempts were made to avoid ready-for-use solutions in the form of “black boxes” due to the significantly limited possibilities of manipulating the functioning of the equipment at the level of the individual analog and digital components.

**Figure 10:**
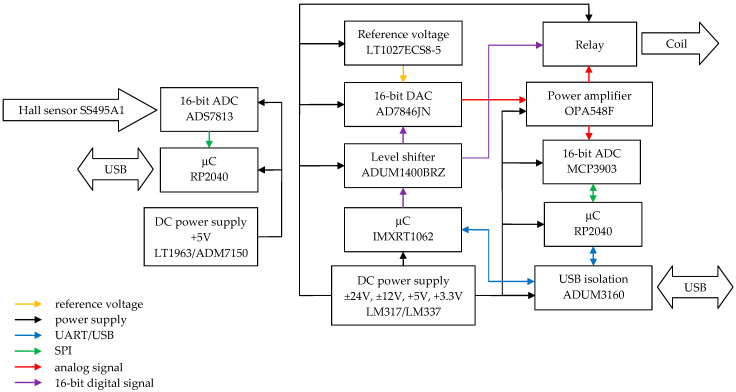
Block diagram of exciter driver.

The control signal generator includes the following functional components (Figure 10): a 16-bit DAC (32 kHz, 40 kHz, 64 kHz, and 80 kHz sampling rates) coupled to a power amplifier controlling the electromagnetic inductor coil; a 16-bit ADC (40 kHz sampling rate) with a Hall sensor providing information about the position of the inductor table; a set of mating 32-bit microcontrollers with external flash memories that operates the DAC and ADC and provides communication with external control software; an internal calibration circuit with an artificial load (for calibration purposes); and power supply circuits. The main component of the measurement data acquisition system is a microcontroller that handles the data transmission protocols of the accelerometers under test with digital outputs. The microcontroller also provides communication with external control software via UART.

The control software consists of two computer applications, controlling the operation of the exciter and the measurement data acquisition system. The software was developed specifically for the test stand and is being improved all the time. Additionally, data processing was verified using Matlab scripts specially developed for this purpose.

**Figure 11:**
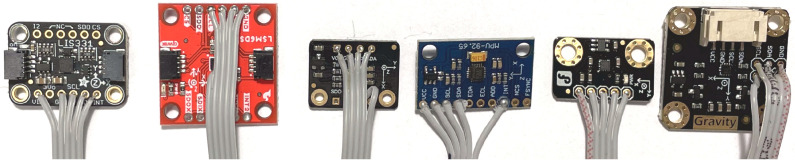
Example modules with MEMS accelerometers.

All tests were conducted in a laboratory room with constant temperature and humidity. Because accelerometers emit heat during their operation, each measurement was preceded by an hour’s annealing of the current sensor under test. In addition, due to the MEMS being mounted on PCBs of different sizes (Figure 11), weight differences were compensated for with additional weights to keep the mass inertia of the system constant (the control consisted of, among other things, recording the position of the table loaded with the sensor under test).

The configuration of the sensors for stage II testing included only the operating range and sampling frequency. On some models, it was necessary to enable maximum resolution mode and disable energy-saving mode. All other features assumed default values. Table 5 summarizes the information on the basic configuration of the selected sensor models.

## 3. Results

### 3.1. Stage I: Results

Table 6 summarizes the values of the largest Allan variances to classify all the sensors tested. Figure 12, Figure 13, Figure 14, Figure A1, Figure A2, Figure A3, Figure A4, Figure A5 and Figure A6 (Appendix A) show the results of AV analysis for accelerometers grouped into three grades: A, B, and C.

In terms of the criterion adopted, the best sensor turned out to be the IIM42352, manufactured by TDK InvenSense. As can be seen, all the tested sensors belonging to the LSM6DS family of accelerometers (STMicroelectronics) are in grade A. It was decided to choose the best of them, i.e., the one with the lowest average AV_2_ value—LSM6DSVX16. Other sensors worthy of attention are the KX132 (Kionix) and ISM330DHCX (STMicroelectronics), which rank third and fourth, respectively, in the accepted classification. Taking into account the previously selected representative of the LSM6 family, the next three in the ranking were selected for further testing, namely, MMA8452Q (NXP Semiconductors), LIS2DW12 (STMicroelectronics), and BMI270 (Bosch Sensortec), and one, the best of grade B: ADXL313 (Analog Devices). It should be mentioned that grade B (ADXL345) and C (MPU6050) sensors are already successfully used in structural health monitoring systems for buildings (see [40]), which, due to their price and performance, can be an interesting alternative to the commonly used expensive solutions.

**Figure 12:**
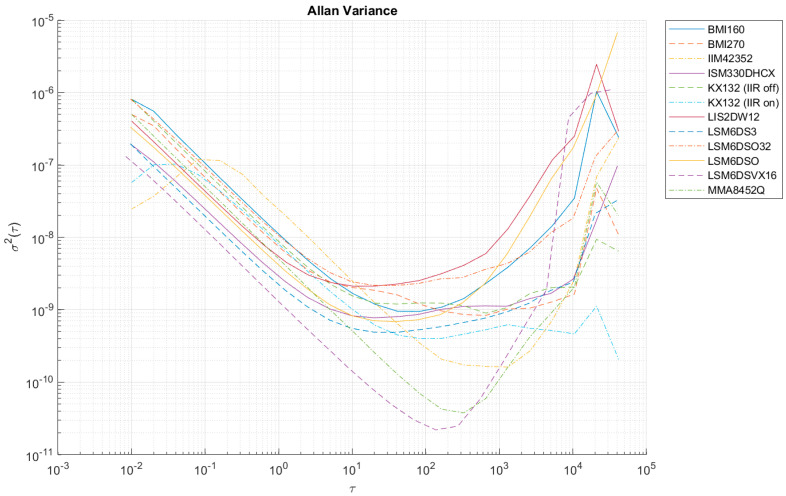
Allan variance for grade A accelerometers (x-axis).

**Figure 13:**
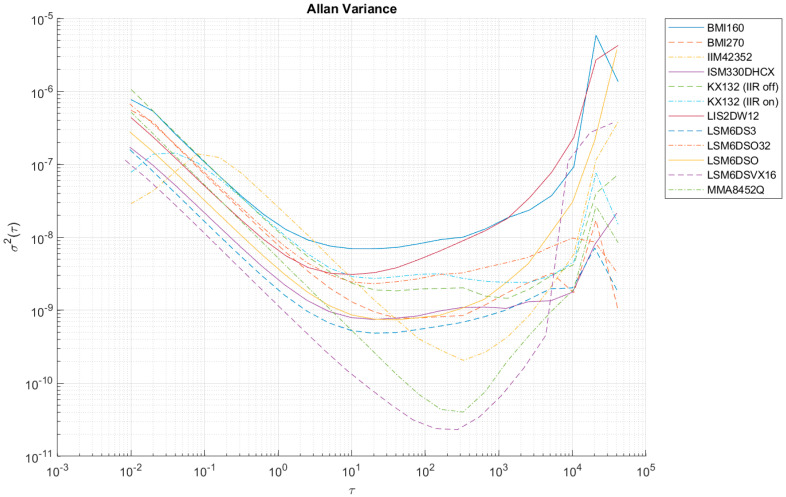
Allan variance for grade A accelerometers (y-axis).

**Figure 14:**
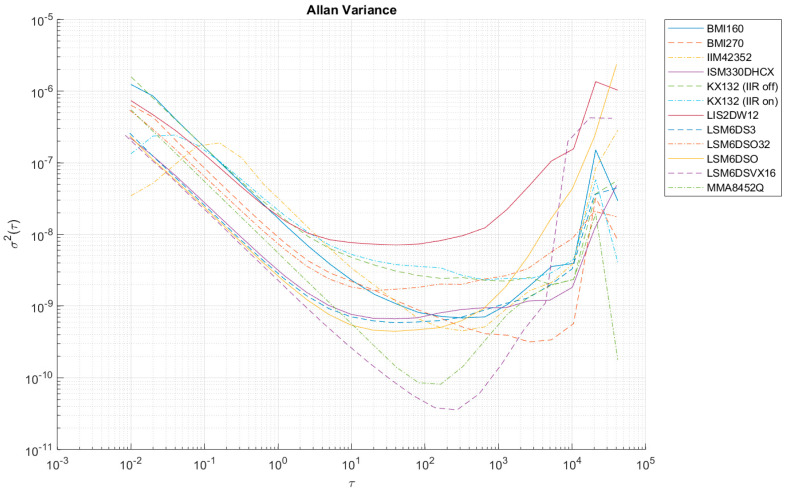
Allan variance for grade A accelerometers (z-axis).

At high sampling frequencies during AV analysis (i.e., 100 Hz), it is possible to register an increase in the AV in the initial parts of the Allan function waveform (e.g., in the case of IIM42352 or KX132), which is the result of filters that effectively reduce the intrinsic quantization noise of the sensors (compare KX132 with the IIR filter on and off—Figure 12, Figure 13 and Figure 14). These filters are enabled in the default configuration of the manufacturers of these sensors, and only in the case of the KX132 IIR filter can they be disabled.

### 3.2. Stage II: Results

In sub-stage S, the sensors were subjected to sinusoidal vibrations, paying attention to the minimum amplitude value that could be recorded by them (see Figure 15, Figure 16, Figure A7, Figure A8, Figure A9, Figure A10, Figure A11, Figure A12, Figure A13, Figure A14, Figure A15, Figure A16, Figure A17, Figure A18, Figure A19 and Figure A20—Appendix B). Two series of tests (with initial amplitudes of 40 and 10 mm/s^2^) are aimed at a preliminary and detailed determination of the threshold value of the vibration amplitude, which can be easily identified visually on the graphs.

Sub-stage P of testing involved sensor loads characterized by an abrupt change in their position with decreasing amplitude. Figure 17, Figure 18, Figure A21, Figure A22, Figure A23, Figure A24, Figure A25, Figure A26, Figure A27, Figure A28, Figure A29, Figure A30, Figure A31, Figure A32, Figure A33 and Figure A34 (Appendix C) show the results of this part of the analysis.

## 4. Interpretation of Results and Discussion

### 4.1. Interpretation of the Results of Stage I

While AV analysis is an extremely important tool in the diagnostics of various types of MEMS sensors, their testing under simulated laboratory conditions fully reflects their properties, which are key features of their suitability for practical use in well-defined environmental conditions. For this reason, a more important issue is the minimum threshold and selectivity of accelerometers in terms of vibrations, which can pose a threat to the monitored construction objects. Despite this, the AV analysis carried out successfully allows preliminary identification of the most promising candidates, which in the case currently under consideration have significantly different resolutions and resulting minimum thresholds (see Table 5). The aforementioned differences should become clearly apparent in the results of the principle tests performed using the proposed procedure.

### 4.2. Interpretation of the Results of Stage II, Sub-Stage S

All analyses were carried out in the Matlab environment, developing scripts with interpretive procedures implemented. All steps in the analysis of the measurement data obtained from the tests are presented below.

- Extracting the clean signal from the raw data.

Obtaining a usable signal from the raw data was carried out using a Butterworth low-pass filter, which has maximally flat amplitude characteristics in the frequency response. A 10th-order filter with a cutoff frequency of 10 Hz was used. Data filtering was carried out in windows of width equal to the number of samples recorded in 1 s. Examples of waveforms of the cleaned signal superimposed on the raw signal are shown in Figure 19 and Figure 20.

- Determination of the noise and signal-to-noise ratio (SNR)

The determination of the SNR is necessary due to the stated goal of this research: this measure relating signal power to noise power is the initial parameter forming the basis for estimating the minimum threshold of the accelerometers under study, understood here as a practical acceleration amplitude threshold identifiable in the raw signal without the use of sophisticated numerical tools. Figure 21 and Figure 22 show comparisons of the SNR values for the sensors tested.

It is rather obvious that the evaluation of sensors on the basis of the SNR alone is neither sufficient nor easy to implement—a good example is the fluctuation of SNR values for signals with the lowest amplitudes in the case of the KX132 accelerometer, which is most likely due to the interference having a form very close to that of the useful signal. (compare Figure 21 and Figure A13). It is worth noting that this situation, although most pronounced for the Y-axis, is repeated for the other axes as well (measurements for each axis were made independently of each other and repeated several times).

- Determination of the modified SNR, MSNR.

Determination of the MSNR value, representing the relation of the power of the cleaned signal to the power of the extracted noise, greatly facilitates individual and comparative analyses of the practical capabilities of the studied sensors. Having functional waveforms with positive and negative values, it is much easier to adopt a clear criterion that can be used to select the most valuable devices. Of course, such a criterion will always be subjectively adopted, being adequate for the expected purpose of each sensor. Figure 23 and Figure 24 show a comparison of the MSNR for the studied sensors.

- Determination of the ENOB.

In the case of S1 and S2 signals, only S2 gives a chance to determine an effective number of bits of practical use for a direct event identification method (i.e., without the use of advanced signal processing methods). This is because in the case of S1 signals, the SNR level is too low to give ENOB values even just approaching unity. Figure 25 compares the ENOB values for the tested accelerometers. Obviously, the achievement of ENOB = 1 for the IIM42352, ISM330DHCX, KX132, and LSM6DSVX16 sensors for the largest amplitudes in the X and Y axes clearly indicates the possibility of identifying an event based on observing the value of only one LSB in the raw data. Undoubtedly, this is an extremely advantageous situation from the point of view of the complexity of embedded systems design and software. Of course, the use of even simple filters based on, for example, a moving average can significantly shift the threshold of identifiable acceleration amplitudes toward much smaller values.

On the other hand, ENOB values should be determined taking into account the full information about the interference, so not only the noise but also the distortion of the signal itself is usually taken into consideration (SINAD). However, taking into account the fact that in the practical applications of the studied sensors the shape and distortion of the recorded forcing signals are not important, the step of determining the level of distortion was omitted.

- Determination of threshold amplitudes based on ENOB and MSNR.

**Figure 16:**
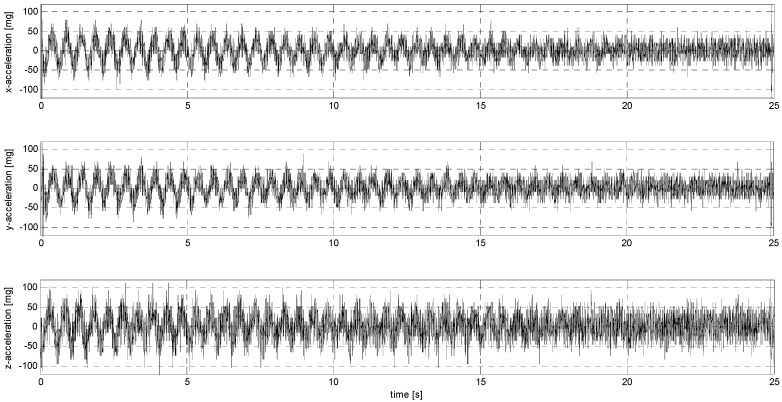
S2 signal recorded by ADXL313.

Analyzing the results of the second stage of testing, it can be seen that all eight sensors can successfully be used to detect sinusoidal vibrations with low frequencies and amplitudes expressed in terms of acceleration located in the range of 20–40 mm/s^2^ (Figure 16, Figure A8, Figure A10, Figure A12, Figure A14, Figure A16, Figure A18 and Figure A20). Particularly noteworthy are the IIM42352, ISM330DHCX, LSM6DSVX16, and KX132 sensors, whose data enable detection of vibrations with smaller acceleration amplitudes reaching up to 10 mm/s^2^ (see Figure A9, Figure A11, Figure A13 and Figure A17, initial data fragment). However, it should be noted that the sensors respond differently when the vibration is continuous in nature with decreasing amplitude and the signal of interest is in the middle of the waveform (see the 19th second in Figure A20) and when the signal is freshly excited (compare with the beginning of the vibration waveform in Figure A19).

The determination of the minimum threshold, understood in this case as a practical characteristic that determines the suitability of the sensor under specified operating conditions, is based on subjective assumptions. An example is the threshold of minimum amplitude, for which the ENOB value is equal to 1. In the case of the IIM42352, ISM330DHCX, LSM6DSVX16, and KX132 sensors, this threshold is about 28 mm/s^2^, 45 mm/s^2^, 46 mm/s^2^, and 44 mm/s^2^, respectively (Figure 25, x-axis), while for the other sensors, it cannot be determined. Another example of a criterion for determining the minimum threshold can be the assumption that, using simple averaging filters, the identification of a signal will be unambiguous as long as its MSNR value is not negative. The minimum amplitude thresholds determined under the above assumption are shown in Table 7.

In addition, given the maximum range of accelerations for which the sensors were configured (2 g), the dynamic range (DR) of each sensor can be determined for the adopted criterion. A comparative summary is shown in Figure 26.

### 4.3. Interpretation of the Results of Stage II, Sub-Stage P

**Figure 17:**
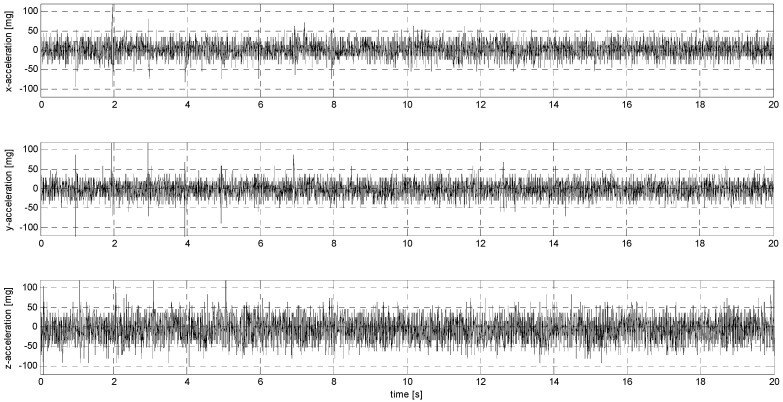
P1 signal recorded by ADXL313.

**Figure 18:**
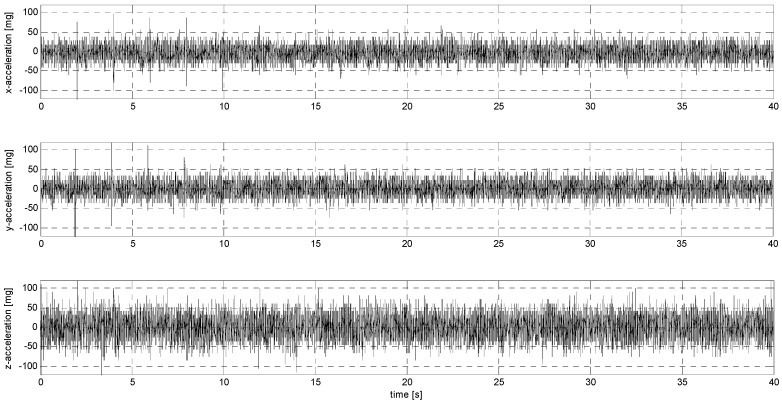
P2 signal recorded by ADXL313.

**Figure 19:**
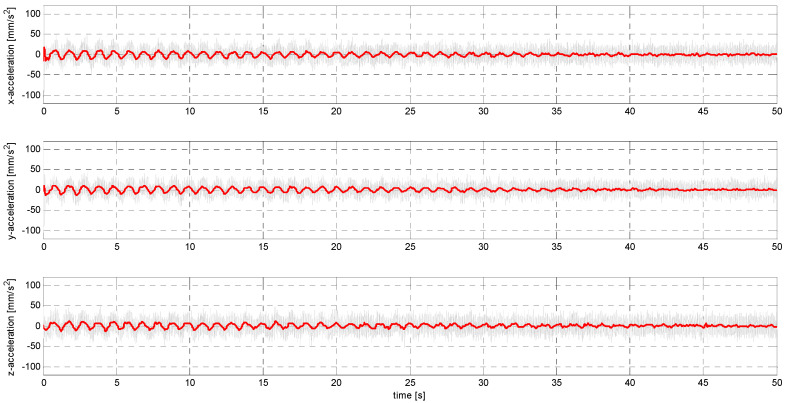
Filtered S1 signal recorded by LSM6DSVX16.

**Figure 20:**
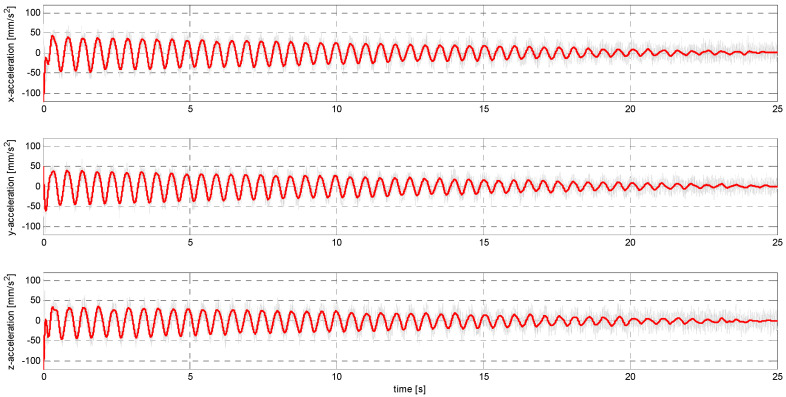
Filtered S2 signal recorded by LSM6DSVX16.

**Figure 21:**
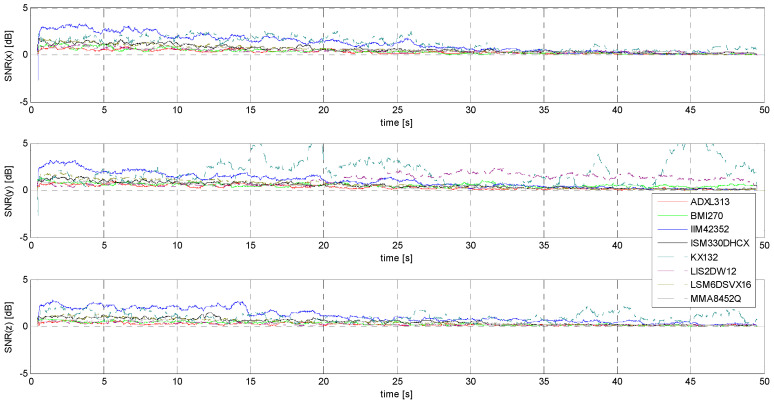
SNR for S1 signal.

**Figure 22:**
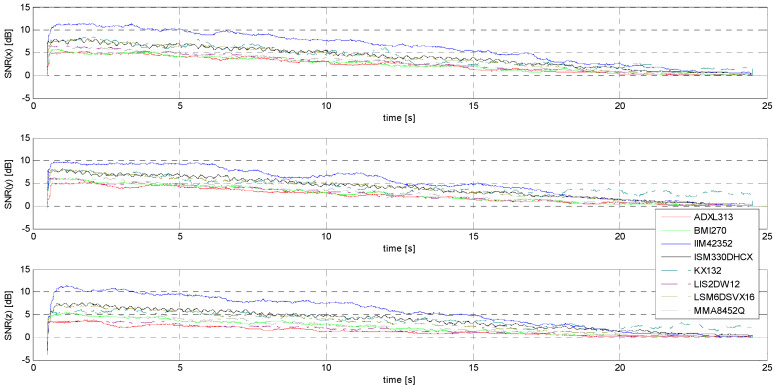
SNR for S2 signal.

**Figure 23:**
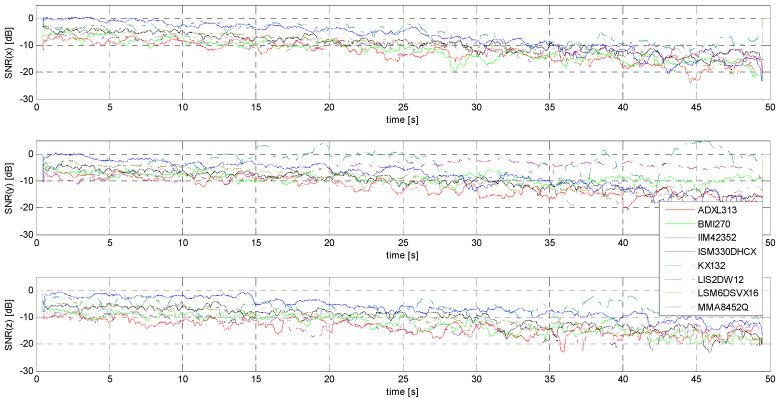
MSNR for S1 signal.

**Figure 24:**
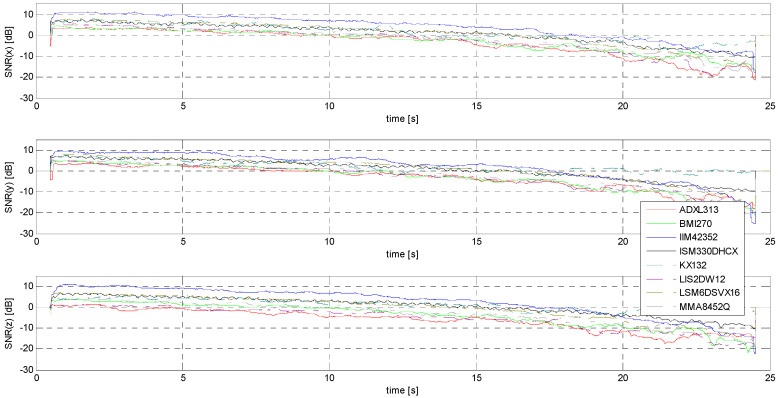
MSNR for S2 signal.

**Figure 25:**
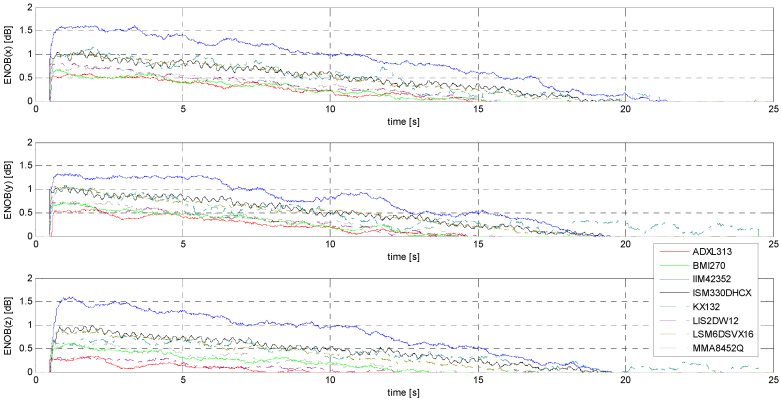
ENOB evaluated for S2 signal.

**Figure 26:**
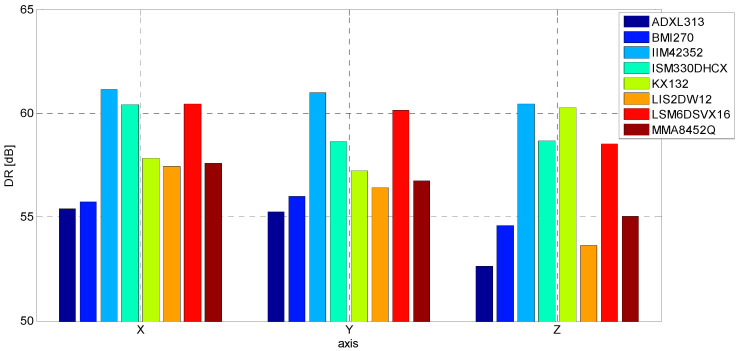
DR evaluated for S2 signal.

**Figure 27:**
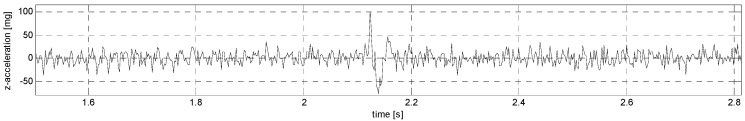
P1 signal recorded by LSM6DSVX16.

**Figure 28:**
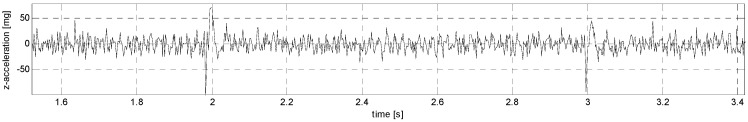
P2 signal recorded by LSM6DSVX16.

**Figure 29:**
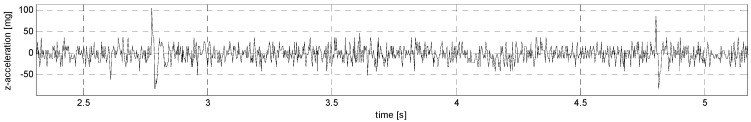
P2 signal recorded by MMA8452Q.

The analysis of the results of recording harmonic signals presented in the previous section is completely different from the case where the goal is to detect disturbances that are of short pulse nature. Obviously, the maximum signal frequency that can be correctly recorded or reproduced by a sampling system must satisfy the Nyquist–Shannon theorem, that is, to accurately reproduce an analog signal using digital samples, the sampling frequency must be at least twice the highest signal frequency. Given that the test used single acceleration pins of about 100 mm/s^2^, an attempt was made to determine the impact of, among other things, the inertia of the MEMS system itself and its ability to record vibrations of much higher frequency and much shorter duration relative to the sampling frequency. The tests were repeated five times, and the results indicate that most accelerometers (all but the KX132, Figure 17, Figure 18, Figure A21, Figure A22, Figure A23, Figure A24, Figure A25, Figure A26, Figure A29, Figure A30, Figure A31, Figure A32, Figure A33 and Figure A34) reproducibly detect impulse shocks with a minimum amplitude of about 70 mm/s^2^ (see Figure 27, Figure 28 and Figure 29).

It should be noted that the configuration of all accelerometers subjected to testing included the activation of only the most basic functions, leaving all others in the default settings provided by the manufacturer. Thus, it can be concluded that in the case of KX132, in the basic configuration of its operation, the default IIR filter effectively eliminates individual pulses by treating them as incidental noise (in the default settings, it is a low-pass filter with a cutoff frequency of 44.4 Hz, when the sampling rate is set to 400 Hz). So a sure solution to this problem is to disable this filter or increase the sampling frequency, but this involves increased power consumption and is also associated with more intensive processing of more information by the microcontroller managing the chip. On the other hand, the low-noise mode of the IIM42352 sensor did not interfere with the recording of signals having the characteristic of single pulses (see Figure A23 and Figure A24). This is because the module under analysis (DK-42352) is equipped with a microcontroller that operates the sensor and preprocesses the raw data (SAM G55).

**Figure 30:**
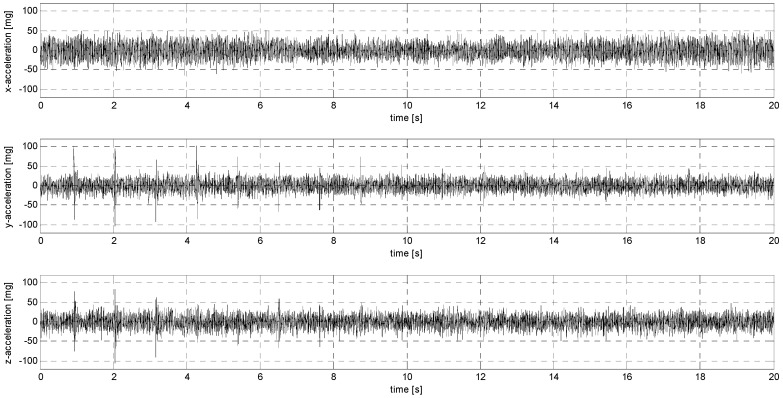
P1 signal recorded by ISM330DHCX (y-axis excitation; pulses recorded in the z-axis).

Since MEMS positioning is very delicate, and considering mounting on low-cost circuit boards, the MEMS sensitivity axis may not be perfectly parallel to the vibration application axis. Transverse motions then appear, which can be registered, but only at significant amplitudes of acceleration forced on the main axis. Such a phenomenon was registered, for example, in the case of the ISM330DHCX sensor (see Figure 30). Note that no such phenomenon was observed for S1/S2 signals.

A final point is the power consumption of the MEMS studied: all draw less than 1 mA from the power source, which makes them extremely attractive in terms of battery-powered embedded systems. Of course, taking into account all the results of the analyses performed, three of the sensors can certainly be considered as potential candidates for the construction of low-cost systems for monitoring objects at risk of low-amplitude shocks—these are the IIM42352, ISM330DHCX, and LSM6DSVX16.

## 5. Conclusions

**Figure 31:**
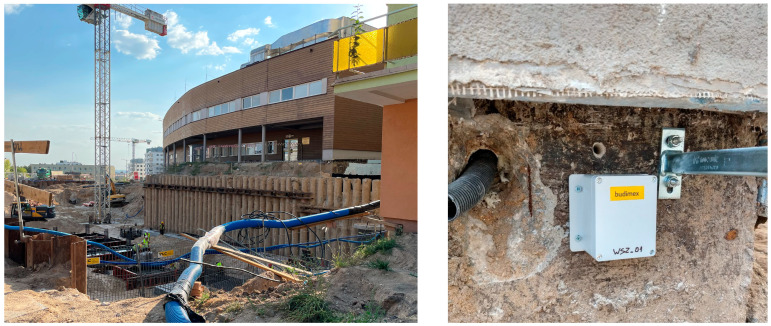
Construction of the Trauma Center of the Regional Specialized Hospital in Olsztyn (Poland); event recorder module installed on the wall of the hospital building adjacent to the construction site.

**Figure A8:**
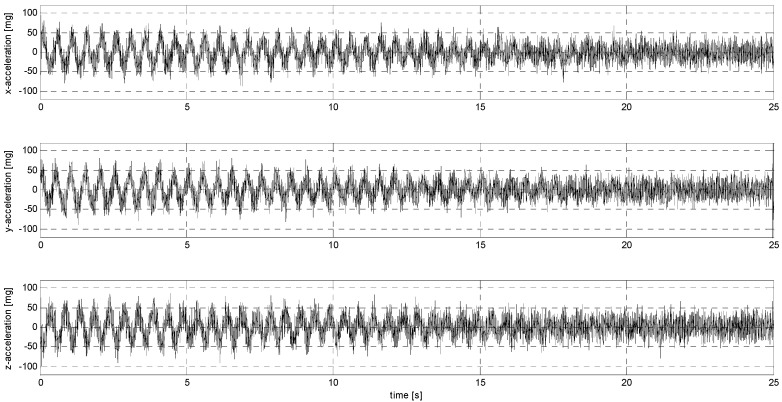
S2 signal recorded by BMI270.

**Figure A9:**
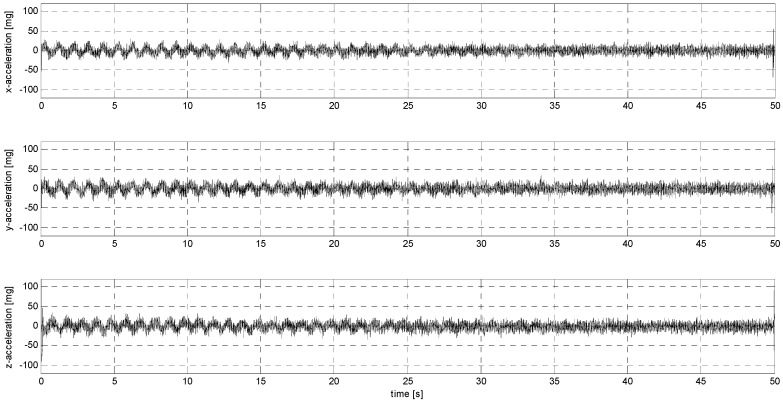
S1 signal recorded by IIM42352.

**Figure A10:**
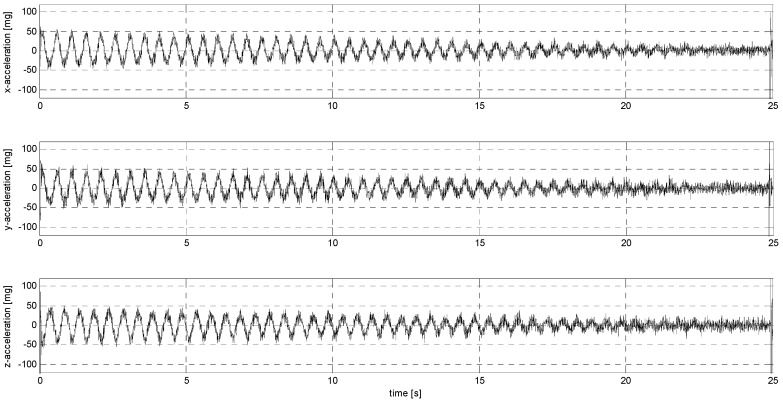
S2 signal recorded by IIM42352.

**Figure A11:**
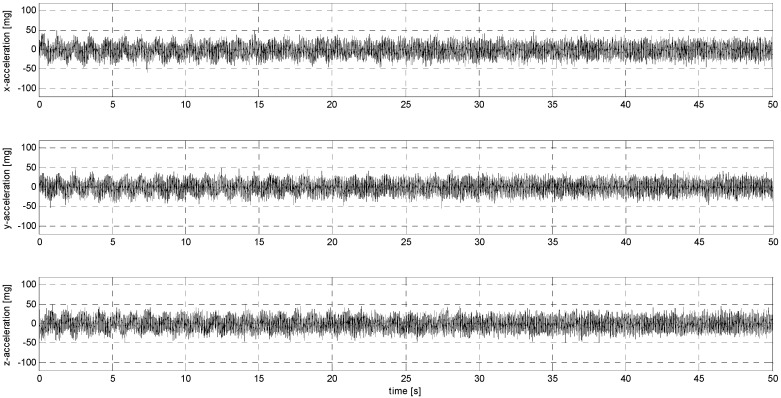
S1 signal recorded by ISM330DHCX.

**Figure A12:**
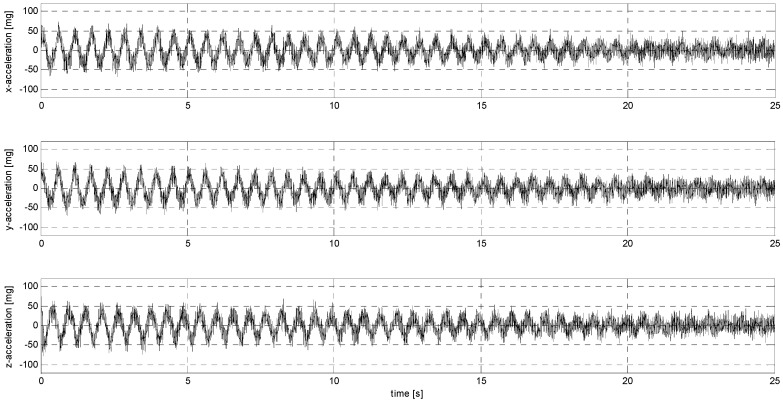
S2 signal recorded by ISM330DHCX.

**Figure A13:**
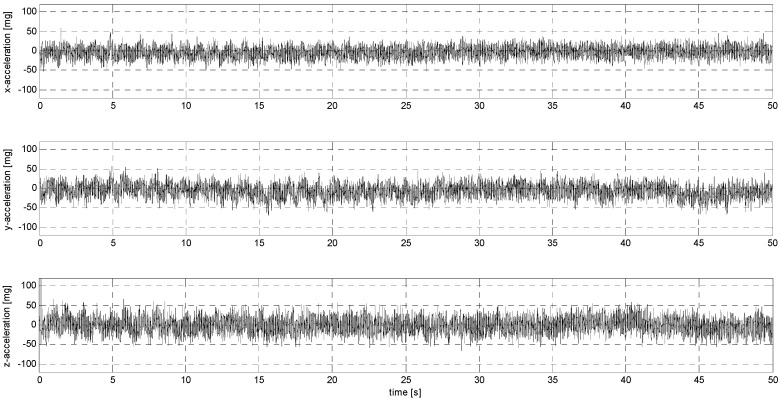
S1 signal recorded by KX132.

**Figure A14:**
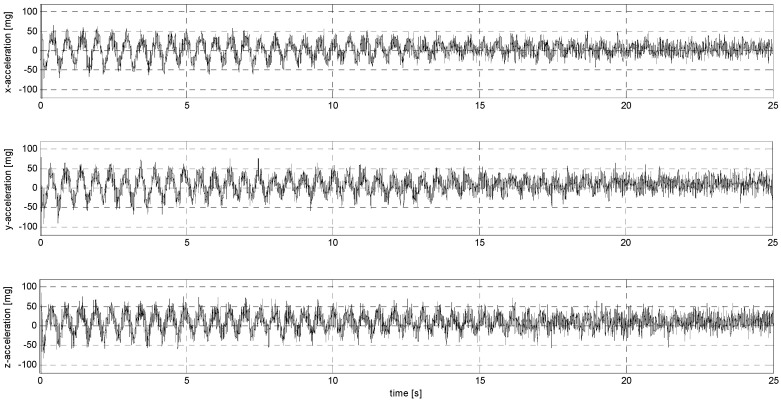
S2 signal recorded by KX132.

**Figure A16:**
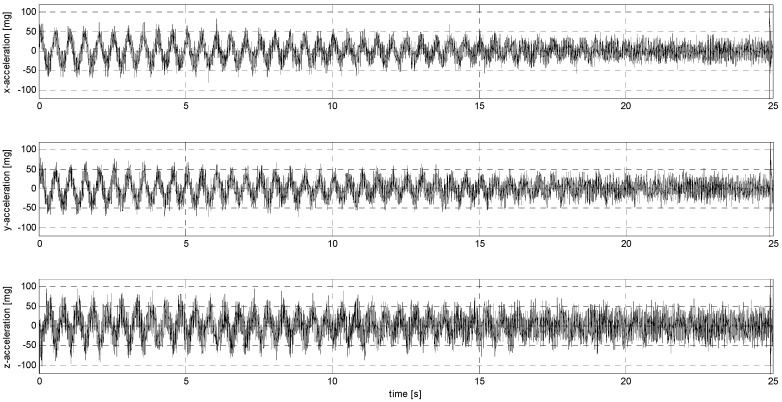
S2 signal recorded by LIS2DW12.

**Figure A17:**
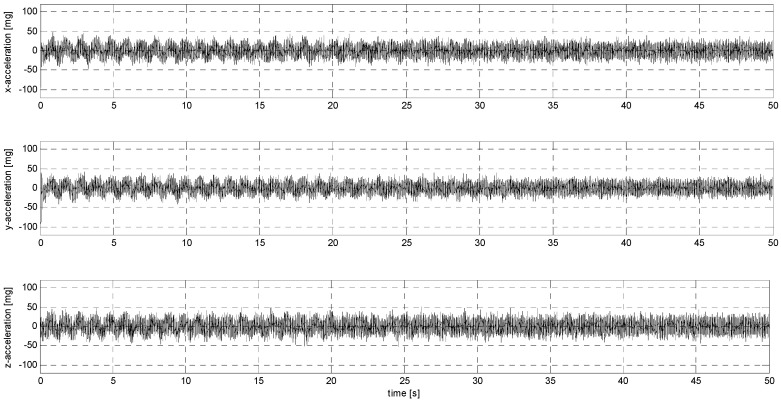
S1 signal recorded by LSM6DSVX16.

**Figure A18:**
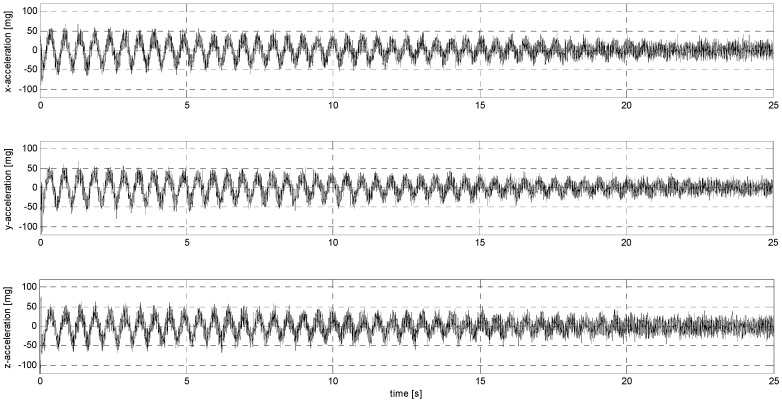
S2 signal recorded by LSM6DSVX16.

**Figure A19:**
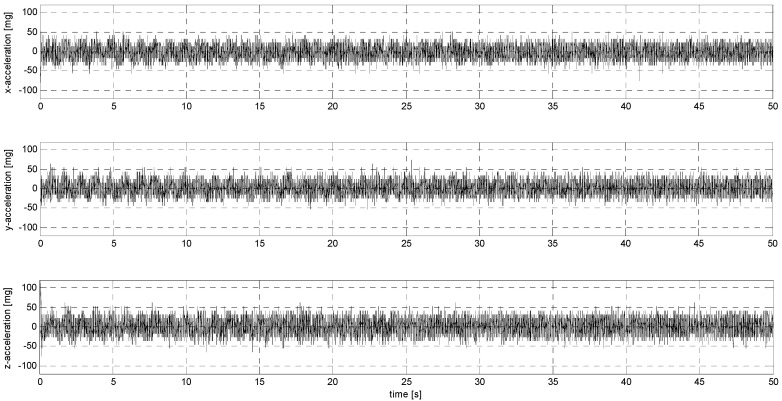
S1 signal recorded by MMA8452Q.

**Figure A20:**
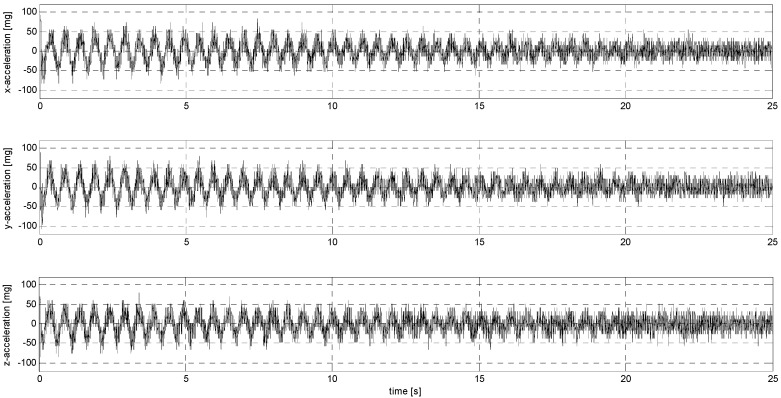
S2 signal recorded by MMA8452Q.

**Figure A21:**
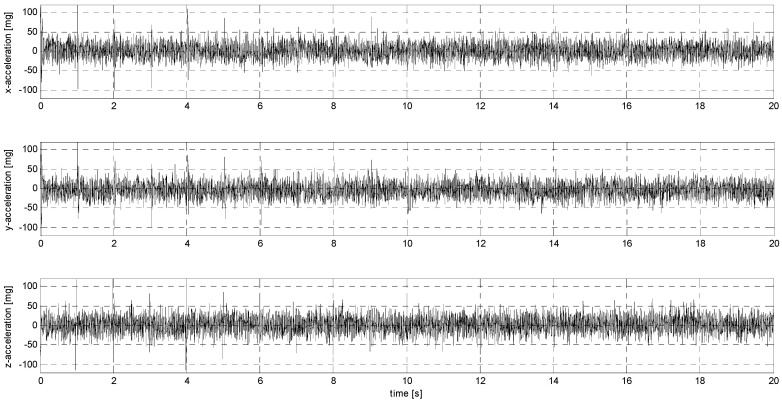
P1 signal recorded by BMI270.

**Figure A22:**
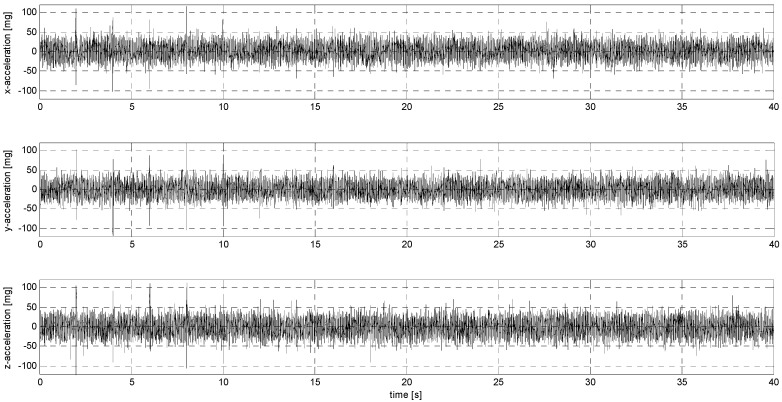
P2 signal recorded by BMI270.

**Figure A23:**
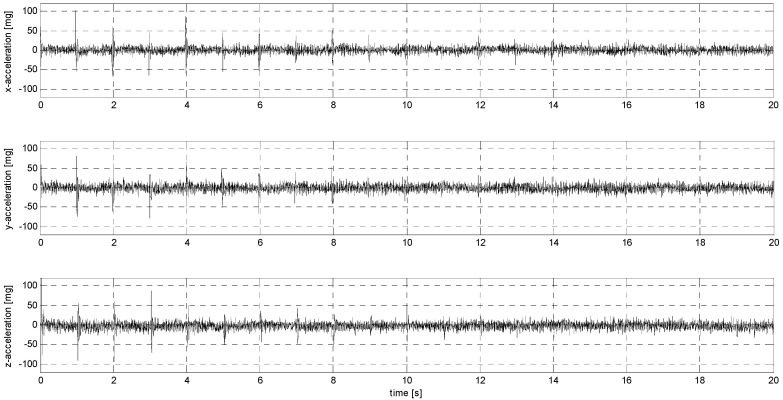
P1 signal recorded by IIM42352.

**Figure A24:**
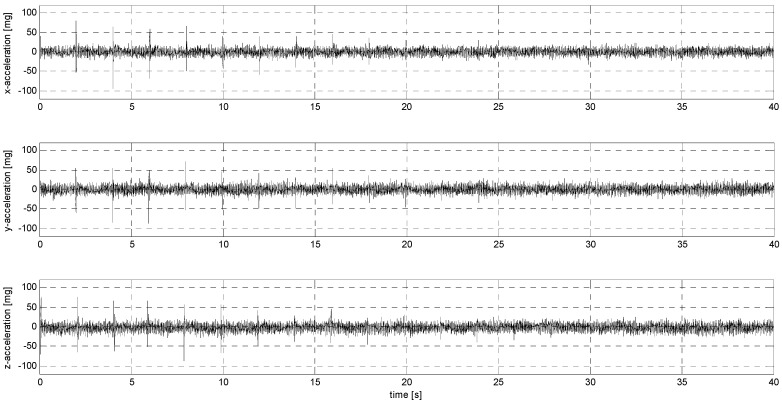
P2 signal recorded by IIM42352.

**Figure A25:**
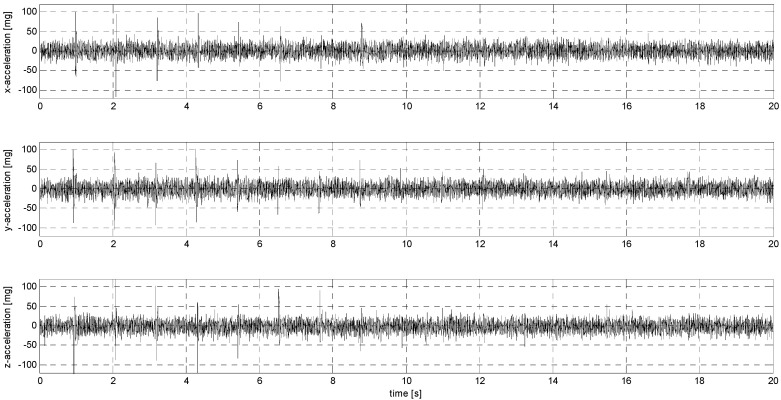
P1 signal recorded by ISM330DHCX.

**Figure A26:**
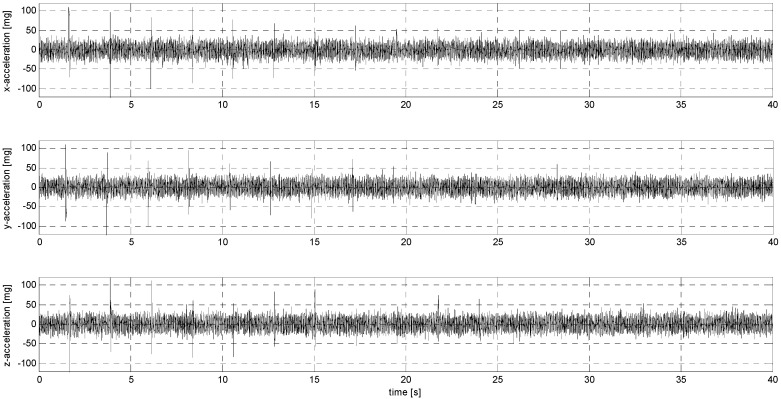
P2 signal recorded by ISM330DHCX.

**Figure A29:**
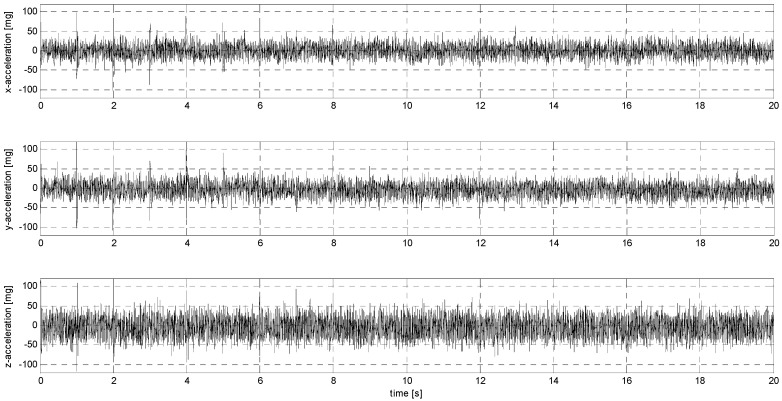
P1 signal recorded by LIS2DW12.

**Figure A30:**
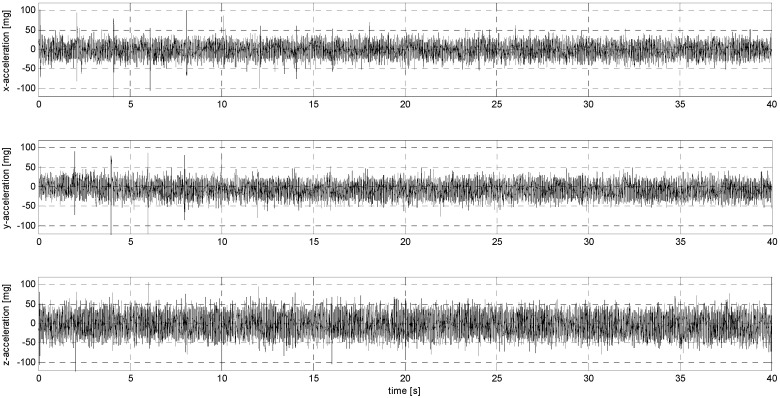
P2 signal recorded by LIS2DW12.

**Figure A31:**
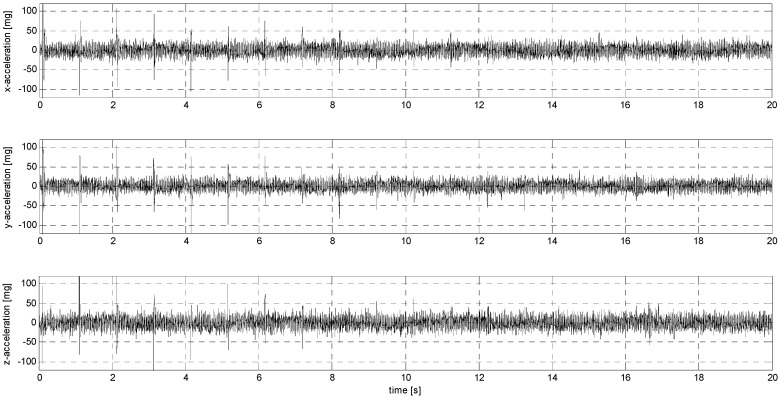
P1 signal recorded by LSM6DSVX16.

**Figure A32:**
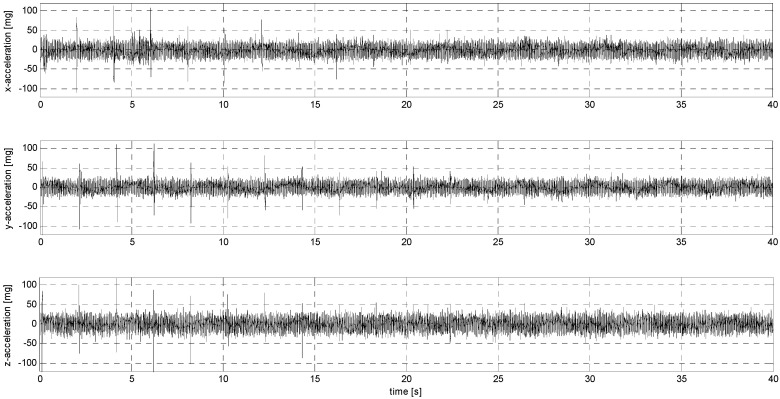
P2 signal recorded by LSM6DSVX16.

**Figure A33:**
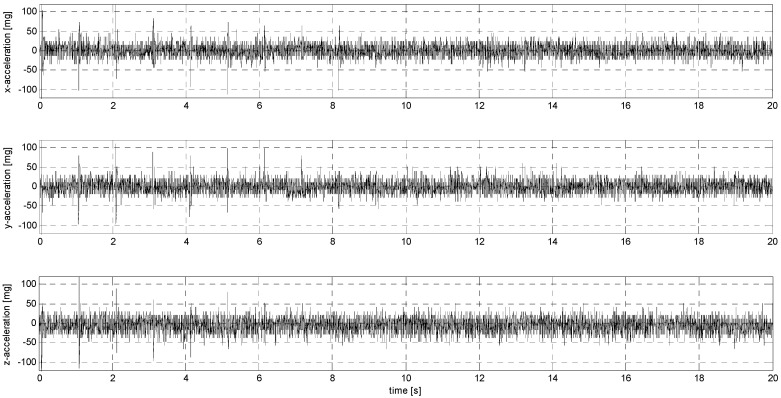
P1 signal recorded by MMA8452Q.

**Figure A34:**
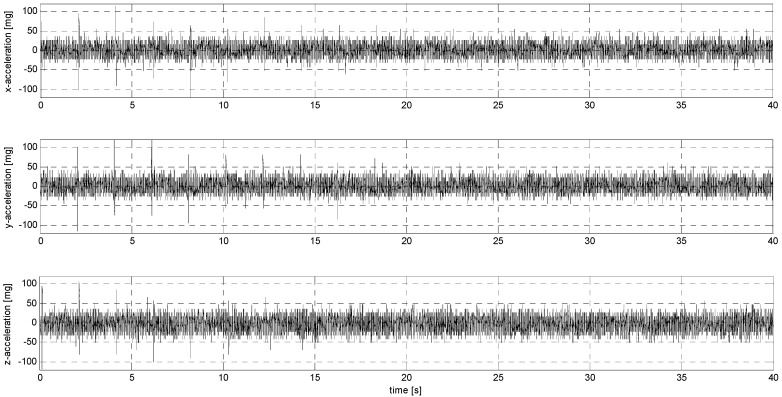
P2 signal recorded by MMA8452Q.

This study proposes a specific set of test methods for MEMS accelerometers in terms of their selection for vibration monitoring of building structures that are threatened by loads characterized by low frequencies and small acceleration amplitudes. Although such threats are quite rare (e.g., in the case of operating rooms in hospitals, not far from which, for example, driven pile foundations are installed, see Figure 31), the emerging possibility of low-cost MEMS sensors creates a huge opportunity for the spread (e.g., in the construction industry) of uncomplicated, and therefore low-failure, as well as cost-effective systems that can effectively warn of such incidents. The following conclusions are drawn from this study:

1. The selection of sensors can be successfully carried out by means of tests involving the loading of the tested sensors with:
  - Harmonic vibrations of linearly varying amplitude;
  - Vibrations of an impulsive nature;
2. The proposed test procedures require the use of precise measurement equipment dedicated to this type of testing (exciters/shakers and data acquisition systems);
3. The application of the proposed test procedure makes the process of interpretation of the obtained results relatively simple, although it requires the adoption of criteria depending on the purpose of the MEMS sensors under analysis;
4. The results of the analysis showed that in the wide range of commonly available low-cost MEMS accelerometers, it is possible to select at least a few models characterized by satisfactory operating stability, high sensitivity, and energy efficiency (e.g., IIM42352, ISM330DHCX, LSM6DSVX16, or KX132);
5. An analysis based solely on the catalog parameters of the sensors does not give a complete picture of the properties and suitability of the sensors for specific purposes; an example is sensors with lower resolutions, which can be successfully used in vibration monitoring systems in construction facilities (e.g., MMA8452Q).

It should be noted that this study did not use embedded DSP systems built into the sensors, including those with AI algorithms. However, such extensions should not be underestimated, as they open up new possibilities and fields of application (in addition to those dedicated by manufacturers, such as fitness trackers, wirstbands, smart watches, earbuds, ankle bands, neck bands, smart clothes, and augmented and virtual reality glasses and controllers). Some of the sensors tested (e.g., MPU6050, MPU6500, and ADXL345) have already been successfully used by the authors in systems for tracking the effects of dynamic loads on buildings, and the last mentioned sensor has even been used in advanced laboratory studies of dynamic soil properties (see [41]). Currently, based on the results of the analyses presented in this study, four modules using MEMS accelerometers have been designed and constructed to monitor the vibration of the hospital building structure near to which foundation work is underway (Figure 31).

The control of vibration and dynamic motion plays an important role in many industrial processes, seismic measurements, navigation and positioning systems, and natural and environmental hazard studies. In these applications, data quality is a priority. Evaluation of sensor data quality depends on the established metrology infrastructure, such as the calibration of digital sensors at nodes, metrological treatment of the entire sensor network, and remote self-calibration. When considering energy-efficient and low-cost accelerometers as measuring devices, it is necessary to trace the calibration method of digital sensors in metrological terms [42]. A calibration system developed at the National Institute of Metrological Research allows the evaluation of the main and transverse sensitivity of three-axis accelerometers by means of single-axis vibration excitation, using inclined planes rigidly attached to a vibration table [42,43,44], and the calibration procedure is based on comparison to a reference transducer (similar to ISO 16063-21 [45]). Calibration of the frequency response is essential to extract the correct information from the measurements. The team of Shimoda at al. [46] developed a synchronized data acquisition system for digital and analog signals and implemented it in the main calibration system for accelerometers. Seeger and Bruns [47] undertook the development of a dynamic calibration of modern sensors with integrated data sampling and digital output for measuring mechanical quantities, such as force, pressure, torque, angular velocity, or acceleration. However, the system is presented with an emphasis on the basic calibration of accelerometers. Taking into account the context of low-cost accelerometers adopted in the presented research, an effort was made to present a low-cost device design enabling a relatively simple methodology for testing low-cost accelerometers in terms of their selection for applications related to the construction industry.

The tests and their results presented in this paper are part of research work involving the construction of embedded, low-cost measurement and monitoring systems, whose components, in addition to MEMS accelerometers, include temperature sensors, GNSS modules, WiFi, and GSM transmission modules, managed by RP2040 32-bit controllers. These systems are built for the needs of fire department rescue units (actions in structures damaged by gas explosions), as well as construction companies engaged in deep foundation work in urban areas.

## Figures and Tables

**Figure 15:**
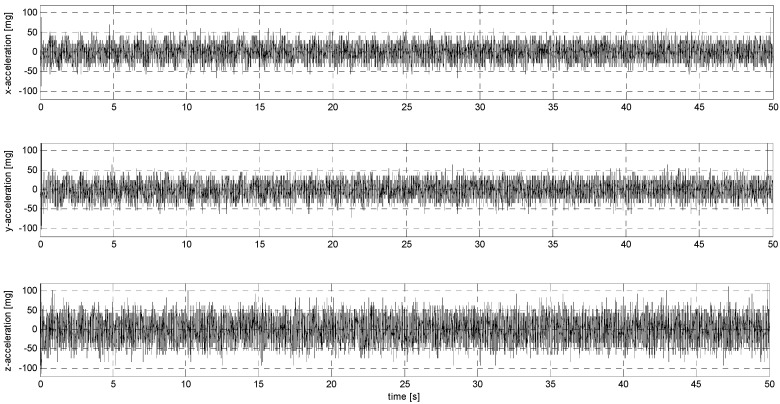
S1 signal recorded by ADXL313.

**Table 1:** The most important parameters of the analyzed accelerometers.

Name	Acc. Range [±g]	Resolution [bits]	Sensitivity [LSB/g]	Max. Output Data Rate [Hz]	Max. Bandwidth [Hz]	Nonlinearity [±%FS]	Current Consumption [mA]	Oper. Temp. Range [°C]	Approx. Cost * [$]
ADXL313	0.5:4.0	10:13	128:1024	3200	0.5 DR ***	0.5	0.300	−40:105	21.38
ADXL343	2.0:16.0	10:13	32:256	3200	0.5 DR	0.5	0.140	−40:85	8.16
ADXL345	2.0:16.0	10:13	32:256	3200	0.5 DR	0.5	0.140	−40:85	3.80
BMA220	2.0:16.0	5:8	2:16	1000	32:1000	2.0	0.250	−40:85	6.04
BMA400	2.0:16.0	11	128:1024	800	0.48 DR	0.5	0.015	−40:85	9.55
BMI160	2.0:16.0	16	2048:16,384	3200	0.22:0.43 DR	0.5	0.600	−40:85	4.16
BMI270	2.0:16.0	16	2048:16,384	1600	0.43:0.46 DR	0.5	0.970	−40:85	17.82
H3LIS331DL	100:400	12	49:195	1000	0.5 DR	2.0	0.370	−40:85	17.20
IIM42352	2.0:16.0	16	2048:16,384	32,000	4000 (1650 **)	0.1	0.280	−40:105	32.39
ISM330DHCX	2.0:16.0	16	2048:16,384	6667	0.5 ODR	n.d.	0.430	−40:105	30.05
KX132	2.0:16.0	16	2048:16,384	25,600	4200 (2900 **)	0.5	0.148	−40:105	15.97
LIS2DH	2.0:16.0	8:12	1:192	5376	n.d.	n.d.	0.185	−40:85	6.54
LIS2DW12	2.0:16.0	16	128:4096	1600	0.5 DR	n.d.	0.090	−40:85	5.46
LIS3DH	2.0:16.0	16	1000:12,000	1250	0.5 DR	n.d.	0.011	−40:85	7.55
LIS331HH	6.0:24.0	16	3000:12,000	1000	0.5 DR	n.d.	0.250	−40:85	14.95
LSM6DS3	2.0:16.0	16	2048:16,384	6664	0.5 DR	n.d.	1.250	−40:85	6.94
LSM6DSO	2.0:16.0	16	2048:16,384	6664	0.5 DR	n.d.	0.550	−40:85	15.88
LSM6DSO32	4.0:32.0	16	1024:8196	6664	0.5 DR	n.d.	0.550	−40:85	17.32
LSM6DSVX16	2.0:16.0	16	2048:16,384	7680	0.5 DR	n.d.	0.650	−40:85	23.96
MMA7660FC	1.5	6	21.33	120	n.d.	n.d.	0.294	−40:85	10.86
MMA8452Q	2.0:8.0	8, 12	256:1024	800	0.5 DR	n.d.	0.165	−40:85	18.44
MPU6050	2.0:16.0	16	2048:16,384	1000	n.d.	0.5	0.500	−40:85	17.65
MPU6500	2.0:16.0	16	2048:16,384	4000	n.d.	0.5	0.450	−40:85	2.48
MSA311	2.0:16.0	12	128:1024	1000	500	2.0	0.130	−40:85	6.54

* price as of October 18, 2024; ** for z-axis; *** DR—output data rate.

**Table 2:** Configuration of data rates.

Name	Data Rate [Hz]	Name	Data Rate [Hz]	Name	Data Rate [Hz]
ADXL313	100	LIS2DW12	100	IIM42352	100
ADXL343	100	LIS3DH	100	ISM330DHCX	104
ADXL345	100	LIS331HH	100	KX132	100
BMA220	125	LSM6DS3	104	LIS2DH	100
BMA400	100	LSM6DSO	104	MMA8452Q	100
BMI160	100	LSM6DSO32	104	MPU6050	100
BMI270	100	LSM6DSVX16	120	MPU6500	100
H3LIS331DL	100	MMA7660FC	120	MSA311	125

**Table 3:** Parameters of experiments conducted in the second stage of this study.

Sub-Stage	Signal	Frequency/ Duration	Maximum Amplitude	Number of Cycles/Steps
S1	sinus	1.0 Hz	10 mm/s^2^	50
S2	sinus	2.0 Hz	40 mm/s^2^	50
P1	step	1 s	0.005 mm	20
P2	step	2 s	0.005 mm	20

**Table 4:** The parameters of the exciter.

Drive System	Impedance [Ohm]	Max Power [W]	Max Amplitude [mm]	Frequency Range [Hz]
electromagnetic	8	200	±10	0.001:2000

**Table 5:** Basic sensor configuration for stage II testing.

Name	Acc. Range [±g]	Resolution [bits]	Sensitivity [LSB/g]	Output Data Rate [Hz]	Special Features
ADXL313	2.0	12	1024	400	-
BMI270	2.0	16	16,384	400	-
IIM42352	2.0	16	16,384	200 *	LNM **
ISM330DHCX	2.0	16	16,384	416	-
KX132	2.0	16	16,384	400	IIR
LIS2DW12	2.0	14	4096	400	-
LSM6DSVX16	2.0	16	16,384	480	-
MMA8452Q	2.0	12	1024	400	-

* highest sampling rate supported by the manufacturer’s application; ** low noise mode.

**Table 6:** Maximum Allan variance values corresponding to quantization noise (AV_2_).

Name	X-Axis	Y-Axis	Z-Axis	Mean (AV_0_)	Grade
H3LIS331DL	9.96 × 10^−3^	8.24 × 10^−3^	9.29 × 10^−3^	9.16 × 10^−3^	C
MMA7660FC	1.09 × 10^−3^	1.10 × 10^−3^	8.84 × 10^−4^	1.02 × 10^−3^	
BMA220	1.02 × 10^−4^	3.81 × 10^−6^	7.96 × 10^−5^	6.18 × 10^−5^	
LIS331HH	2.65 × 10^−5^	2.30 × 10^−5^	2.98 × 10^−5^	2.64 × 10^−5^	
MPU6050	9.90 × 10^−6^	9.51 × 10^−6^	1.96 × 10^−5^	1.30 × 10^−5^	
MPU6500	4.49 × 10^−6^	4.48 × 10^−6^	1.22 × 10^−5^	7.06 × 10^−6^	B
ADXL345	5.74 × 10^−6^	3.02 × 10^−6^	8.83 × 10^−6^	5.87 × 10^−6^	
ADXL343	7.68 × 10^−6^	2.76 × 10^−6^	7.03 × 10^−6^	5.82 × 10^−6^	
MSA311	2.75 × 10^−6^	3.03 × 10^−6^	6.21 × 10^−6^	4.00 × 10^−6^	
LIS3DH	2.45 × 10^−6^	2.10 × 10^−6^	2.72 × 10^−6^	2.42 × 10^−6^	
LIS2DH	2.27 × 10^−6^	2.04 × 10^−6^	2.62 × 10^−6^	2.31 × 10^−6^	
BMA400	1.02 × 10^−6^	1.01 × 10^−6^	2.53 × 10^−6^	1.52 × 10^−6^	
ADXL313	6.34 × 10^−7^	7.26 × 10^−7^	1.77 × 10^−6^	1.04 × 10^−6^	
BMI160	8.07 × 10^−7^	7.72 × 10^−7^	1.24 × 10^−6^	9.39 × 10^−7^	A
LSM6DSO32	8.17 × 10^−7^	6.73 × 10^−7^	5.40 × 10^−7^	6.77 × 10^−7^	
BMI270	4.99 × 10^−7^	5.52 × 10^−7^	6.34 × 10^−7^	5.62 × 10^−7^	
LIS2DW12	4.03 × 10^−7^	4.38 × 10^−7^	7.35 × 10^−7^	5.26 × 10^−7^	
MMA8452Q	4.99 × 10^−7^	5.19 × 10^−7^	5.49 × 10^−7^	5.22 × 10^−7^	
LSM6DSO	3.33 × 10^−7^	2.79 × 10^−7^	2.38 × 10^−7^	2.83 × 10^−7^	
LSM6DS3	1.96 × 10^−7^	1.60 × 10^−7^	2.59 × 10^−7^	2.05 × 10^−7^	
ISM330DHCX	1.95 × 10^−7^	1.72 × 10^−7^	2.25 × 10^−7^	1.97 × 10^−7^	
KX132 (IIR on)	1.02 × 10^−7^	1.43 × 10^−7^	2.42 × 10^−7^	1.63 × 10^−7^	
LSM6DSVX16	1.31 × 10^−7^	1.14 × 10^−7^	2.43 × 10^−7^	1.63 × 10^−7^	
IIM42352	1.17 × 10^−7^	1.28 × 10^−7^	1.78 × 10^−7^	1.41 × 10^−7^	

**Table 7:** Amplitude thresholds for MSNR ≥ 0.

Name	X-Axis [mm/s^2^]	Y-Axis [mm/s^2^]	Z-Axis [mm/s^2^]	Resolution [bits]	Threshold [mm/s^2^/LSB]
ADXL313	33.98	34.59	46.65	12	9.77
BMI270	32.73	31.68	37.25	16	0.61
IIM42352	17.52	17.85	19.01	16	0.61
ISM330DHCX	19.05	23.42	23.34	16	0.61
KX132	25.71	27.54	19.39	16	0.61
LIS2DW12	26.88	30.21	41.74	14	2.44
LSM6DSVX16	18.98	19.68	23.69	16	0.61
MMA8452Q	26.43	29.11	35.45	12	9.77

**Figure A1:**
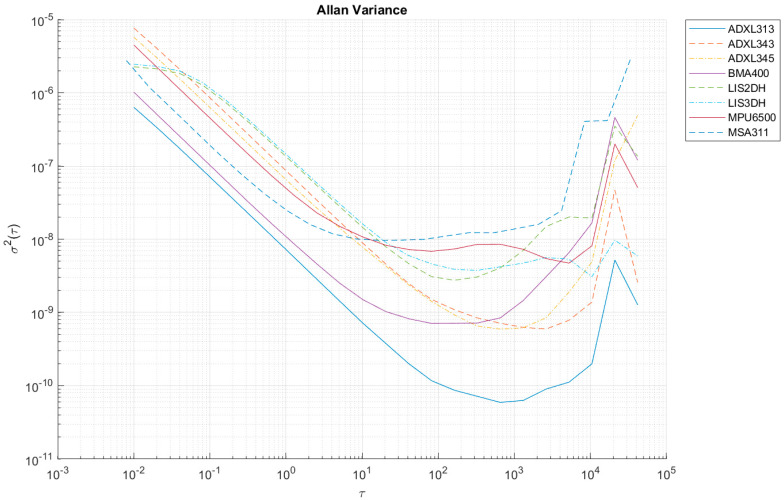
Allan variance for grade B accelerometers (x-axis).

**Figure A2:**
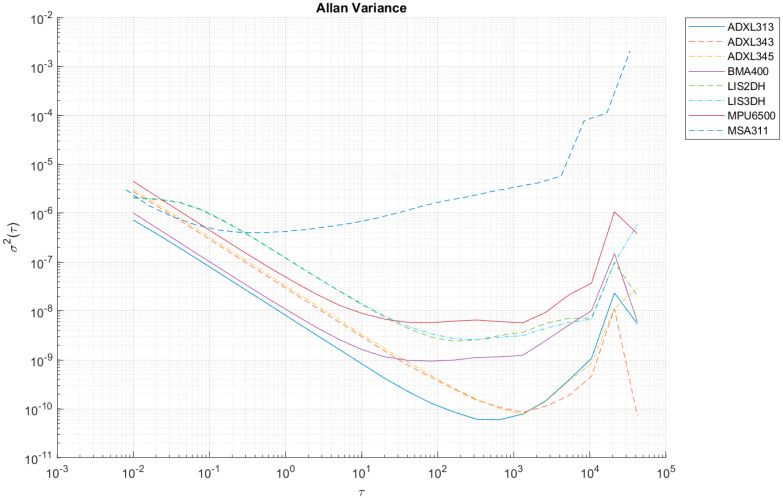
Allan variance for grade B accelerometers (y-axis).

**Figure A3:**
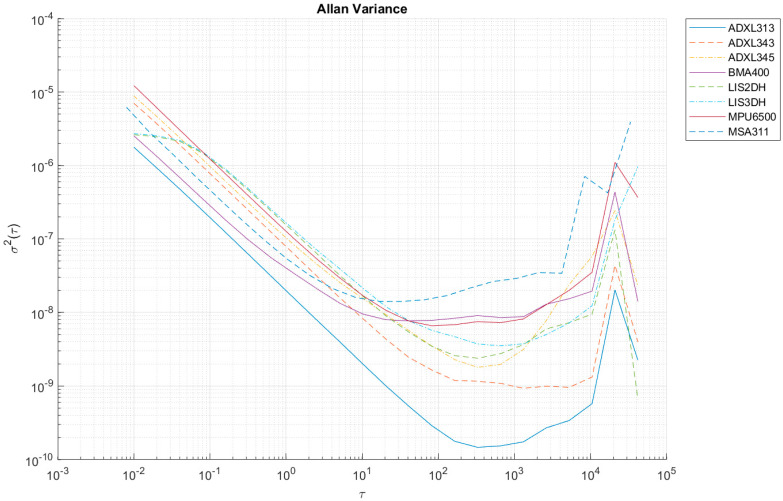
Allan variance for grade B accelerometers (z-axis).

**Figure A4:**
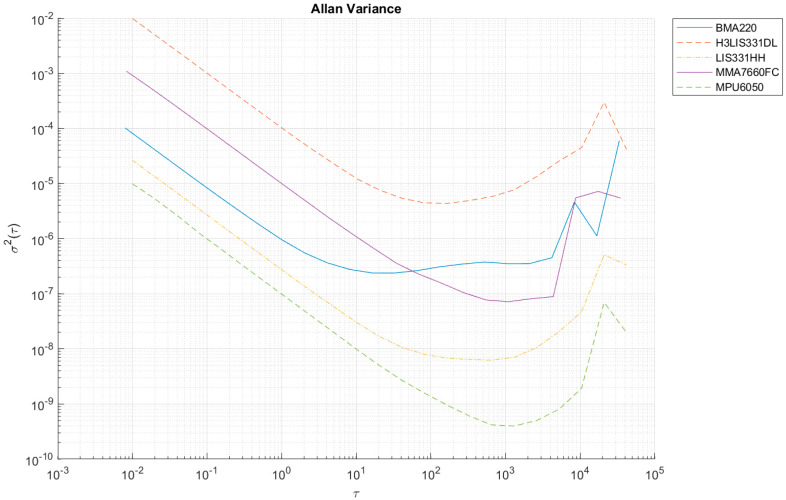
Allan variance for grade C accelerometers (x-axis).

**Figure A5:**
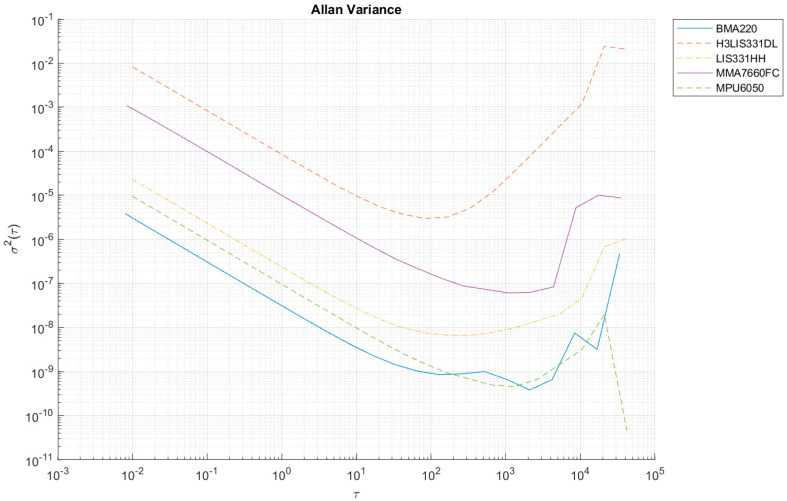
Allan variance for grade C accelerometers (y-axis).

**Figure A6:**
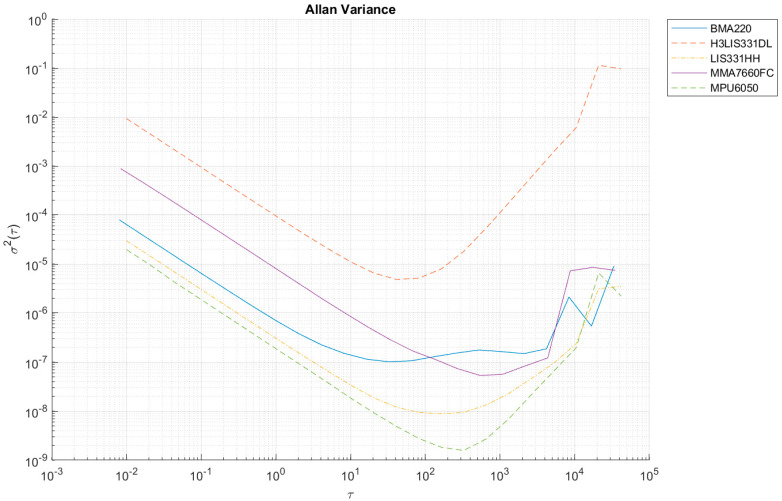
Allan variance for grade C accelerometers (z-axis).

**Figure A7:**
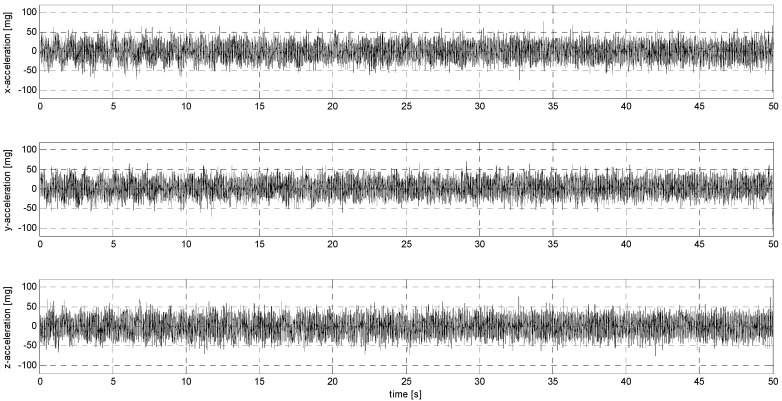
S1 signal recorded by BMI270.

**Figure A15:**
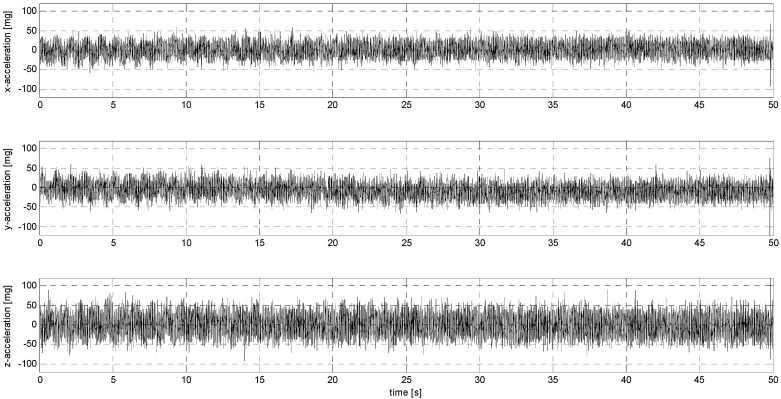
S1 signal recorded by LIS2DW12.

**Figure A27:**
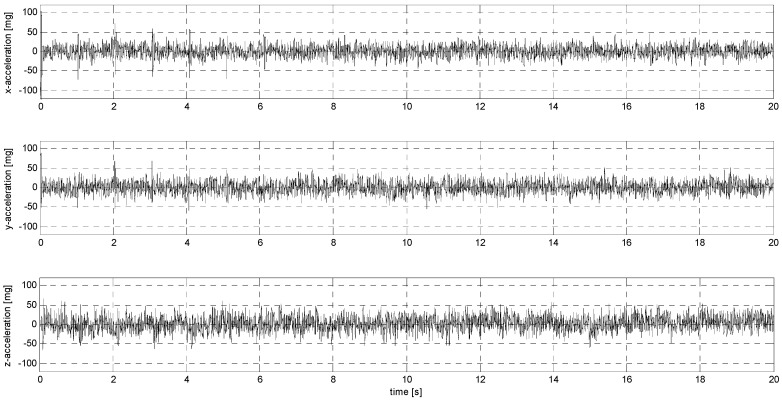
P1 signal recorded by KX132.

**Figure A28:**
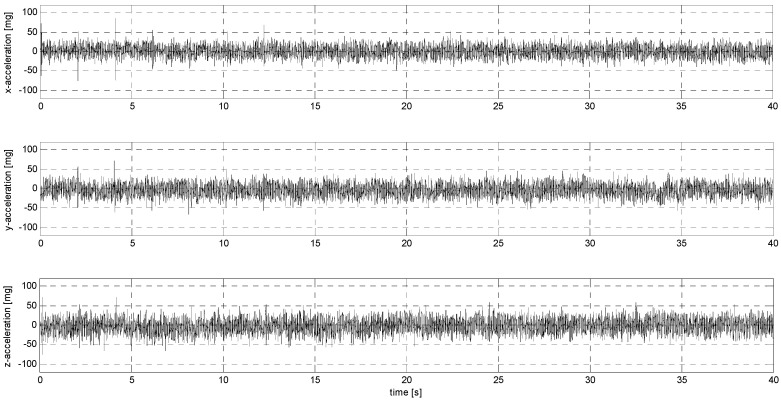
P2 signal recorded by KX132.

## Data Availability

The data that support the findings of this study are available from the corresponding author, [P.S.], upon reasonable request.
